# Neutral and adaptive drivers of genomic change in introduced brook trout (*Salvelinus fontinalis*) populations revealed by pooled sequencing

**DOI:** 10.1002/ece3.8584

**Published:** 2022-02-07

**Authors:** Brent Brookes, Hyung‐Bae Jeon, Alison M. Derry, John R. Post, Sean M. Rogers, Shelley Humphries, Dylan J. Fraser

**Affiliations:** ^1^ 5618 Department of Biology Concordia University Montréal QC Canada; ^2^ Département des sciences biologiques Université du Québec à Montréal Montréal QC Canada; ^3^ 2129 Department of Biology University of Calgary Calgary AB Canada; ^4^ Parks Canada Natural Resource Management Branch Radium Hot Springs BC Canada

**Keywords:** adaptive differentiation, genetic diversity, introduced species, neutral diversity, *Salvelinus fontinalis*, whole‐genome sequencing

## Abstract

Understanding the drivers of successful species invasions is important for conserving native biodiversity and for mitigating the economic impacts of introduced species. However, whole‐genome resolution investigations of the underlying contributions of neutral and adaptive genetic variation in successful introductions are rare. Increased propagule pressure should result in greater neutral genetic variation, while environmental differences should elicit selective pressures on introduced populations, leading to adaptive differentiation. We investigated neutral and adaptive variation among nine introduced brook trout (*Salvelinus fontinalis*) populations using whole‐genome pooled sequencing. The populations inhabit isolated alpine lakes in western Canada and descend from a common source, with an average of ~19 (range of 7–41) generations since introduction. We found some evidence of bottlenecks without recovery, no strong evidence of purifying selection, and little support that varying propagule pressure or differences in local environments shaped observed neutral genetic variation differences. Putative adaptive loci analysis revealed nonconvergent patterns of adaptive differentiation among lakes with minimal putatively adaptive loci (0.001%–0.15%) that did not correspond with tested environmental variables. Our results suggest that (i) introduction success is not always strongly influenced by genetic load; (ii) observed differentiation among introduced populations can be idiosyncratic, population‐specific, or stochastic; and (iii) conservatively, in some introduced species, colonization barriers may be overcome by support through one aspect of propagule pressure or benign environmental conditions.

## Graphical abstract

Understanding the drivers of successful species invasions is important for conserving native biodiversity and for mitigating the economic impacts of introduced species. We investigated neutral and adaptive variation among nine introduced high‐alpine brook trout (*Salvelinus fontinalis*) populations from a common source using whole‐genome pooled sequencing. We found some evidence of bottlenecks without recovery, no strong evidence of purifying selection, and little support that varying propagule pressure or differences in local environments shaped observed neutral genetic variation differences.

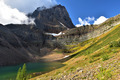

## 1 INTRODUCTION

Accidental and intentional human‐driven introductions of non‐native species are ubiquitous, yet the drivers of successful colonization are rarely known (Hayes & Barry, 2008; Lee, 2002). Invasive species often experience fitness advantages by having fewer predators and inhabiting generalist niches, which can offset inbreeding depression while also facilitating adaptation and plasticity to novel, variable environments (Colautti et al., 2017). Determining consistent predictors of colonization success in association with environmental and genetic factors is particularly challenging, albeit imperative for mitigating species invasions and for improving reintroduction strategies of endangered species (Lee, 2002; Louback‐Franco et al., 2020; Sakai et al., 2001; Sauers & Sadd, 2019).

Although successful species introductions often occur into habitats with familiar environmental conditions (Hayes & Barry, 2008; Moyle & Marchetti, 2006), species may also colonize novel environments when adequate propagule pressure (i.e., number of introduction events and number of individuals introduced) ensures that sufficient genetic variation is available for survival and adaptation (Arismendi et al., 2014; Facon et al., 2006; Duncan, 2011). For example, genetic diversity should be increased through propagule pressure by introducing more individuals and/or by carrying out multiple introductions (Hamilton et al., 2015; Via & Lande, 1985). Introduced species can also become locally adapted to both abiotic and biotic environmental factors (Carroll et al., 2001; Filchak et al., 2000), with a population‐ and context‐specific nature (Briscoe Runquist et al., 2020; Coulson et al., 2017; Schindler & Parker, 2002). Therefore, successful introductions are thought to be dependent on genetic factors associated with both sufficient propagule pressure and adaptive response to the introduced environment (Allendorf & Lundquist, 2003; Prentis et al., 2008). However, studies with the genomic resolution needed to clarify the relative influence of both processes are rare (Dlugosch & Parker, 2008; Narum et al., 2017; Yoshida et al., 2016).

Genomic approaches can improve understanding of how propagule pressure and environmental factors affect genetic diversity during introductions into novel environments (Frachon et al., 2019; Micheletti & Narum, 2018; Narum et al., 2017). Most evolutionary changes at the molecular level, and most of the variation within species, are neutrally evolving due to random genetic drift, gene flow, and bottlenecks (Narum et al., 2017). Conversely, when a genetic variant is putatively adaptive, molecular divergence at loci underlying adaptive traits is maintained by selection, commonly inferred by comparing neutral and adaptive genetic diversity among populations (Dennenmoser et al., 2017; Hamilton et al., 2015; Hecht et al., 2015). Emerging methods such as whole‐genome pooled sequencing (pool‐seq) can capture such genomic variation by sequencing pooled groups of individual DNA samples from the same populations together to characterize single nucleotide polymorphisms (SNPs) throughout the genome (Schlötterer et al., 2014). Pool‐seq is particularly useful to quantify genome‐wide neutral and adaptive variation of introduced species in a cost‐effective manner (Davey et al., 2011; Kurland et al., 2019; Narum et al., 2013; Stanford, 2019).

Socio‐economically important salmonid fishes are among the world's most invasive species and ideal for examining factors influencing colonization success (Krueger & May, 1991; Lecomte et al., 2013; Vigliano et al., 2007). Salmonid invasions resulted from transplants worldwide in over 97 countries for sport‐fishing or through aquaculture escapees into the wild (Fausch, 2007). Hatchery stock management influences the level of genetic diversity in introduced salmonid populations, as stocks with high genetic variability are considered important for introduction success and for avoiding founder effects or bottlenecks (Bert et al., 2007; Kelly et al., 2006). Because environmental factors such as spawning area availability and habitat (stream/lake) size can regulate salmonid abundance (Krueger & May, 1991) and genetic diversity (Bernos & Fraser, 2016; Neville et al., 2009; Rieman & Allendorf, 2001), they may also create conditions for introduced salmonid genotypes under selection to confer fitness advantages and thereby potentially influence colonization success (Benjamin et al., 2007; Hecht et al., 2015; Kinnison et al., 2008).

**FIGURE 1:**
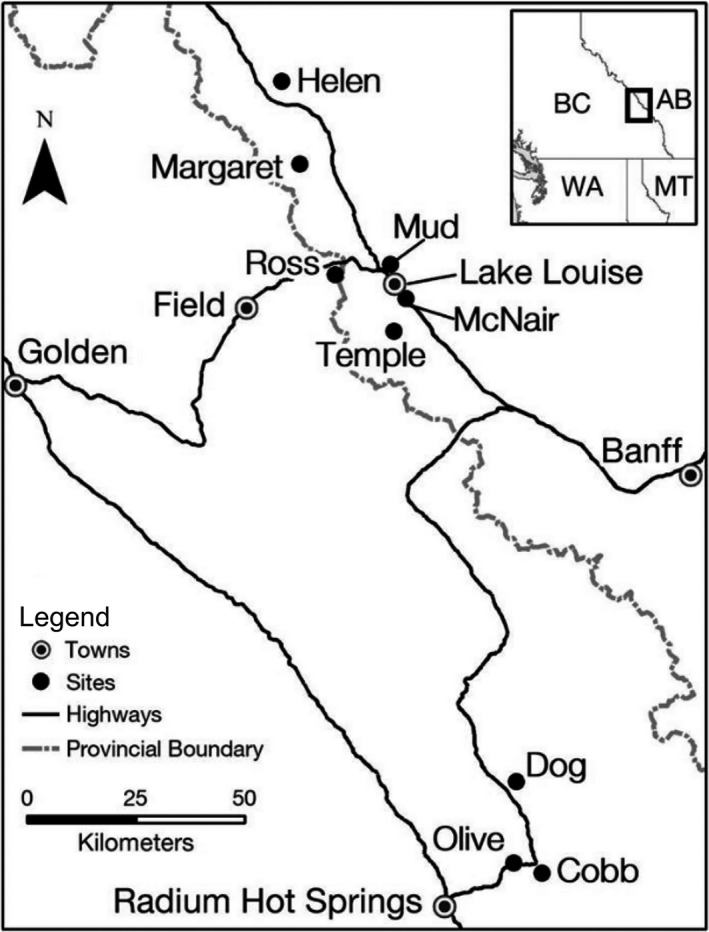
Map of nine sampled lakes for brook trout in their introduced range of Alberta (AB) and British Columbia (BC), Canada

Here, we examine population genomic structuring of nine populations of brook trout (*Salvelinus fontinalis*) established through extra‐limital introductions using SNPs generated from a pool‐seq approach, with the aim to quantify the neutral and adaptive genetic variation associated with successful introduction. To enhance sport‐fishing opportunities, 100,000's of brook trout were stocked between 1941 and 1973 into mountain lakes in several national Parks in the Rocky Mountains of Canada (Figure 1; Table 1), by hatcheries using a hatchery strain originating from the eastern USA (National Parks stocking records). To restore native aquatic ecosystems, Parks Canada has initiated the manual removal of brook trout in several lakes (Pacas & Taylor, 2015). These lakes represent novel environments for brook trout relative to their eastern North American range: high elevations (1,185–2,400 m), covered by ice for 7–9 months of the year (native range 4–9), high pH (7.73–8.45), and variation in spawning site availability due to snowfall runoff among populations (Fassnacht et al., 2018; Power, 1980; Wood et al., 2014; Yates et al., 2019; Table 1). This system of recently introduced populations of the same origin provides a unique opportunity to investigate how salmonids colonized and evolved in different environments outside of their native range, over an average span of approximately ~19 generations (range of 7–41, based on average spawning age; Glaser et al., 2021). Moreover, the study lakes exhibit a wide range of variation in 14 environmental variables and in propagule pressure, from 2,500 to 55,500 individuals introduced and 1–22 introduction attempts (Table 1).

**TABLE 1:** Summarized environmental information for the nine study lakes (2017–2018), along with Parks Canada stocking information for brook trout (1941–1973) and nucleotide diversity (π) within each pool of samples, using 362,493 filtered, biallelic SNPs common to all populations

	Overall Range	Cobb	Dog	Helen	Margaret	McNair	Mud	Olive	Ross	Temple
Abiotic										
Area (ha)	1.66–18.00	2.29	11.50	2.48	18.00	1.66	7.20	1.66	6.61	3.25
Volume (m^3^)	30,135–1,851,206	149,000	316,000	139,621	1,851,206	30,135	217,351	32,833	525,347	156,332
Depth (m)	3.60–28.20	8.00	3.80	15.00	28.20	4.00	7.20	3.60	21.50	14.70
Elevation (m)	1,185–2,400	1,260	1,185	2,400	1,808	1,352	1,600	1,470	1,735	2,207
Number of tributaries	0–2	0	2	1	1	2	2	1	1	1
Upstream discharge (m^3^/s)	0–4,860	0	254	78	4,860	1,054	32	72	509	0
Downstream discharge (m^3^/s)	0–6,251	0	538	186	5,364	974	310	355	6,251	501
Distance from nearest road (m)	5–6,000	2,700	2,600	6,000	5,070	5	1,600	5	3,200	2,000
pH	7.73–8.45	8.03	8.18	8.45	8.03	8.10	8.09	7.73	8.17	7.91
Mean seasonal temperature (°C)	10.00–16.70	16.70	15.30	11.50	11.70	11.60	13.20	10.60	10.00	10.50
Temperature variance (°C)	3.00–9.80	4.30	9.80	4.80	3.00	9.20	9.20	6.50	4.80	6.10
Spawning sites	0–4	0	2	4	2	2	3	2	4	2
Biotic										
Zooplankton density (ind^a^/L)	0.12–17.50	3.52	3.31	2.90	0.12	4.59	5.30	1.40	6.93	17.50
Macroinvertebrate density (ind^a^/m^2^)	53.16–468.75	90.53	257.19	118.54	62.42	72.38	69.41	272.74	468.75	53.16
Jaccard species dissimilarity index	0.40–0.88	0.40	0.88	0.40	0.72	0.79	0.76	0.40	0.40	0.40
Stocking										
Census Size	95–2,650	95	2,140	1,037	1,720	269	1,431	2,416	2,053	2,650
First year stocked	1941–1964	1947	1941	1964	1963	1963	1954	1947	1954	1964
Final year stocked	1963–1973	1973	1972	1965	1963	1968	1968	1972	1967	1968
Number of times stocked	1–22	19	16	2	1	6	10	22	7	3
Total number of fish stocked	2,500–55,500	49,500	55,500	4,000	5,000	2,500	45,050	16,250	24,000	6,000
Mean number of fish stocked per event	416–5,000	2,475 (200–7,000)^b^	3,468 (500–9,000)^b^	2,000	5,000	416 (250–1,000)^b^	4,505 (800–11,000)^b^	706 (200–2,500)^b^	3,428 (1,000–6,000)^b^	2,000
π Male	0.00365–0.01133	0.01133	0.00365	0.00519	0.00666	0.00630	0.00446	0.00610	0.00415	0.00402
π Female	0.00446–0.01652	0.00747	0.00638	0.00868	0.01652	0.00543	0.00611	0.00446	0.00497	0.00463

^a^ Individuals.

^b^ Within lake range.

We hypothesized that standing neutral genetic variation among populations would be positively associated with propagule pressure, while adaptive genetic variation would be associated with environmental variables. Based on the stocking history and environmental data collected from different lakes (Table 1), we predicted that: (i) greater propagule pressure would lead to an increase in neutral genetic variation barring introduction bottlenecks; (ii) neutral genetic variation would be positively correlated with lake (habitat) volume; (iii) postcolonization bottleneck events would be associated with lower neutral genetic variation and lead to a lower proportion of deleterious mutations (Hedrick & Garcia‐Dorado, 2016); and (iv) an increase in putatively adaptive loci would be associated with broad environmental differences among lakes; while signals of adaptive genetic variation would be positively correlated to lake volume and to the relative availability of spawning sites.

## 2 METHODS

### 2.1 Study site

Within Banff, Kootenay, and Yoho national parks, Canada, nine lakes were selected for their physical isolation from other lakes, small size, limited inlet/outlet expanse, and brook trout dominance (Figure 1). According to available records (Donald & Alger, 1984; Parks Canada stocking records), these brook trout originated from a common origin (Paradise Brook Trout Company, Pennsylvania USA). The fish were used to establish broodstocks, originating from 100,000's of eyed eggs, in two Parks Canada hatcheries (Banff and Jasper) for subsequent stocking into park lakes; however, further hatchery information on the number of breeders used in the hatchery and hatchery genetic diversity data was unavailable. Three study lakes (Margaret, Dog, and McNair) are potentially open to seasonal gene flow from brook trout populations residing in adjacent bodies of water through otherwise unpassable outlet waterfalls in extreme weather scenarios (Adams et al., 2000; Thompson & Rahel, 1998; Table 1).

### 2.2 Sampling methods

Sampling of brook trout was conducted in August 2017, using a standardized, mixed‐mesh gill net protocol until 5%–10% of fish in each lake were captured; captured fish were euthanized with an overdose of clove oil following CCAC and Parks Canada‐approved procedures. Caudal fin tissue was collected from each fish and stored in 95% ethanol for DNA extraction, while sex was determined by abdominal dissection. Methodology associated with capture method (i.e., net mesh sizes/lengths, set durations) and census population size estimates obtained in concurrent research and used in analyses below are described in Yates et al. (2021).

To examine whether environmental variables were positively correlated with neutral genetic diversity and adaptive differentiation, 14 abiotic and biotic environmental variables were quantified in each lake between May 2017 and August 2018 (Table 1; Appendix S2). Variables were chosen for their relationship to regulating abundance in salmonids such as habitat size, spawning availability, resource competition, and survivability (i.e., Temperature, pH); winter measurements were not taken due to accessibility (Krueger & May, 1991; National Research Council, 2004). Seep (groundwater upwellings), inlet, and outlet number (tributaries), and water discharge, were measured by circumnavigation of each lake as estimated metrics of spawning site availability. Distance to each lake, considered a stocking variable due to the effort to stock these lakes by hand, was calculated with Parks Canada hike information and Google Earth v9.2.58.1 along hiking trails or directly from the nearest vehicle access based on known roadworks during the stocking periods, which remained unchanged year to year. Connectivity, bathymetry, and lake volume were calculated with ArcGIS version 10.3.1, Google Earth, and obtained from Parks Canada records.

In summer of 2017, depth profiles of temperature, dissolved oxygen, pH, and conductivity were measured twice in each lake at 1 m sequential depths to 0.5 m above bottom with a multiparameter YSI Professional series sonde (model 10102030; Yellow Springs Inc., Yellow Springs, Ohio, USA). HOBO MX2202 Pendant wireless temperature/light dataloggers (Onset, MA USA) were also deployed at the center of each lake and recorded measurements every 30 min at 0.5 m depth from beginning of July to mid‐September. Detailed descriptions of macroinvertebrate and zooplankton sampling are in Appendix S2. Jaccard's dissimilarity index for fish species within each lake was calculated using presence–absence data collected from the sampling period (R package Adespatial, v 0.3‐8; Stéphane Dray et al., 2020); R v 4.0.0 (R Core Team, 2020) and RStudio v 1.2.1335 (RStudio Team, 2020) were used for statistical analyses.

### 2.3 DNA extraction, pooling, and sequencing

DNA extractions from fin tissue were conducted using *Qiagen* blood and tissue kits (Qiagen, Germany) and the manufacturer protocol. To ensure equal quantities of DNA in the pooled samples, DNA quality and quantity were initially assessed by 1% Agarose gel electrophoresis using HindIII digested Lambda DNA run at 100V to assess possible DNA degradation. Multiple quality tests per individual were conducted on a *Qubit* Fluorometer 2.0 (Invitrogen, USA) selecting for quantity >20 ng/µl and confirmed in a *NanoDrop* spectrophotometer (Thermo Scientific, USA) as well as estimates of 260/230 and 260/280 ratios greater than >1.8 quality.

Individual DNA was then pooled by sex, and population (18 total pools) with 20 individuals in each pool; exceptions were Cobb females (*n* = 17) and both McNair sexes (*n* = 8) due to low population and sample sizes. Twenty individuals per pool were chosen to ensure a balance between available population samples and to have equal representation between sexes (two pools of 20 per population), while maintaining accurate unbiased allele frequency estimates (Anand et al., 2016; Boitard et al., 2012). Albeit from different sexes, the adoption of two pools per population also provided a degree of sampling replication for some population genomic analyses, such as genomic‐wide diversity and genetic differentiation. Fifty µl of each individual sample was selected for each pool at a dilution of 10 ng/µl, with DNA concentrations confirmed both prior to and post using a *Qubit* Fluorometer. DNA was pooled together at a final concentration of 3 ng/µl, confirmed using a *Qubit* Fluorometer.

Genomic libraries of these pooled DNA samples were prepared by Génome Québec Innovation Centre, Montréal, Québec, Canada, via a shotgun approach with PCR with *Illuminia TrueSeq* LT adaptors (Illumina, USA). All pools passed quality and quantity requirements and were sequenced each on two lanes of *NovaSeq 6000 S4* flowcell (Illumina) and paired‐end reads of 100 base pairs (bp). Coverage was estimated based on the assumption that the brook trout genome is approximately 3Gb, based on the Animal Genome Size Database (http://www.genomesize.com/).

### 2.4 Pool‐seq pipeline and SNP discovery

A reference genome of charr (*Salvelinus* sp.) available from NCBI (ASM291031v2, Christensen et al., 2018; Genome size = ~2.4 GB, scaffold N50 = 1.02 Mbp, Contig N50 = 55.6 Kbp, masked mapping) was used due to its close phylogenetic and karyotypic relationship with brook trout (Timusk et al., 2011). The reference genome was prepared using Burrows‐Wheeler Aligner (BWA) v 0.7.12 (Li & Durbin, 2009), indexed with SAMtools v 1.5 (Li et al., 2009), and a dictionary was created using Picard tools v 2.17.11 (http://broadinstitute.github.io/picard/, accessed 20‐11‐2019) to permit sequence alignment.

SNP discovery was performed using the PPalign module of the PoolParty pipeline v 0.8 (Micheletti & Narum, 2018); the methods and packages in this module are detailed below. Mapping, alignment trimming, and filtering to the charr reference genome were performed with BWA‐MEM v 0.7.12, SAMtools v 1.5 using a mapping quality threshold of 10, and SAMblaster v 0.1.24 (Faust & Hall, 2014), while filtering for a quality score threshold of 20 was performed by BBMap v 37.93 (Bushnell, sourceforge.net/projects/bbmap/, accessed 20‐11‐2019), and summarized with Fastqc v 0.11.7 (Andrews, 2010). SNP filtering was carried out using common parameters for salmonid species, found below (Horn et al., 2020). Duplicate sequences and unpaired reads were filtered using SAMtools, BBMap, and Picard tools with a minimum fastq trimming length of 25 bp. An indel window of 15 bp was used to mask SNPs around indel regions. SNP calling was facilitated conservatively by BCFtools v 1.5 (Li et al., 2009) with a quality score of 20, a minimum global allele frequency of 0.05, and a minimum global coverage of 10. Raw reads were checked for quality using FastQC and MultiQC v1.7 (Ewels et al., 2016). After SNP calling, multiallelic SNPs that could be paralogs were removed following Létourneau et al., 2018; Narum et al., 2017; Terekhanova et al., 2019. Finally, for all analyses, the PPanalyze module of PoolParty was used to filter out duplicated loci and filter for loci common between all populations with minimum global coverage of 20, maximum global coverage of 100, and minimum allele frequency of 0.05. Of all tested SNPs, the proportion of putatively adaptive loci—that is, loci with greater deviation from average—was negligible (0.15%), and therefore putatively adaptive loci were not removed. Following alignment, *mpileup* files were run through the PPstats module of PoolParty to estimate depth of coverage, alignment statistics, and genome coverage. Collectively, a total of 362,493 SNPs remained that were common among all populations in the dataset (and biallelic, with scaffold removed); these were used for all subsequent genomic analyses, except the Cochran Mantel Haenszel (CMH) tests described below.

### 2.5 Neutral genetic diversity and differentiation

Estimates of nucleotide diversity within each pool were established using PoPoolation 2 (Kofler et al., 2011) with files provided by the PPalign module. We ran PoPoolation 2 with a minimum count of four for the minor allele and minimum coverage of 20 so as not to lose SNPs because a minimum must be met across all pools. We also used a maximum coverage of 100 to remove potentially paralogous regions (Li, 2011). Lastly, we used “sanger” fastq‐type for Phred64, a window and step size of 250, and a pool size represented by 2× the individuals, which is suggested for diploid species. Pairwise estimates of genetic differentiation (using *F*_ST_) between the 18 pools were determined with Poolfstat v 1.1.1 (Hivert et al., 2018) with a minimum read count of two, a minimum coverage per pool of 20, and a maximum coverage per pool of 100, while using the same minor allele frequency as the original file of 0.05 and removing indels, as commonly adopted (Kofler, Orozco‐terWengel, et al., 2011; Micheletti & Narum, 2018).

### 2.6 Relationships between neutral genetic diversity and propagule pressure or environmental variables

For regressions between nucleotide diversity and environmental or stocking variables, a correlation matrix for scaled (with the *scale* function in R) stocking and environmental variables was firstly run to remove potentially correlated variables at a cutoff of 0.7 with the *psych* R package v 1.9.12 (Revelle, 2019). To further remove multicollinearity, we conducted a linear variance inflation (VIF) analysis with all stocking and environmental variables in *car* v 3.0‐5 (Fox & Weisberg, 2019). Both multicollinearity removal techniques were used as VIF analysis was capped at nine variables because of degrees of freedom limitations. The correlation matrix and VIF analysis with a cutoff of 10 (Bagheri & Midi, 2009; Neter et al., 2004) left lake volume, elevation, pH, zooplankton density, macroinvertebrate density, number of tributaries, and total number of fish stocked. Beta‐regressions were then run in RStudio with *betareg* v 3.1‐2 (Cribari‐Neto & Zeileis, 2010) for all remaining variables separately (i.e., nucleotide diversity ~lake volume, nucleotide diversity ~pH); additive and interaction terms were not calculated as we lacked sufficient power with 9 pools per sex (Table S1). Sexes were run separately to avoid pseudo‐independence, as they had identical variable data but different dependent variables (nucleotide diversity). Model visualizations were conducted with ggplot2 v 3.2.1 (Wickham, 2016). All analyses were completed in R.

### 2.7 Deleterious mutations within populations

Before identifying putatively deleterious mutations within the study populations, all variants were annotated using SnpEff v 4.3 t (Cingolani et al., 2012). At the beginning of annotation steps, a genome database was built based on both gff3 and fasta files obtained from NCBI (Agarwala et al., 2018). The deleterious variants were sorted by the following three categories, named for their putative impact: “high,” a variant with a significant deleterious impact on the coding region (e.g., start codon lost, stop codon gained, frameshift variant); “moderate,” a non‐disruptive variant that may affect efficacy (e.g., missense variant, splice region variant); and “low,” an innocuous or unlikely deleterious variant. We do not know the fitness consequences of these deleterious categories for brook trout but aimed to use this test as a method to observe the putative genetic load in each pool. To investigate whether populations experienced inbreeding depression and bottleneck events after introduction, we calculated and compared allele frequencies of putatively deleterious mutations across pools using R script implemented in the PoolParty pipeline along with the filtered, common‐loci dataset, a method used commonly across taxa (Kuang et al., 2020; Mathur & DeWoody, 2021).

We also used lake‐specific, CMH chi‐squared tests to examine statistically differentiated allele frequencies between populations, as well as to infer the number of SnpEff annotated gene products shared and not shared between populations to complement analyses of adaptive differentiation below. The PPanalyze module of the PoolParty pipeline was used to create sync files of each population comparison (male and female pools combined, not filtered for common loci to increase power at picking up gene products). PoPoolation 2 (Kofler et al., 2011) was employed with minimum count of the minor allele is 20, minimum coverage of 20, and maximum coverage of 100. *p*‐values were corrected with an FDR of .05 using Benjamini and Hochberg correction. Identified genes were run through SnpEff to annotate the VCF file from the reference charr genome, and then through BLAST (Altschul et al., 1990) and QuickGO (Binns et al., 2009), to determine gene ontology and function by taking the first available annotated gene product for each BLAST definition. After annotation, SNPs on the scaffold of the vcf file were removed, leaving only biallelic SNPs aligned to linkage groups of the charr reference genome.

### 2.8 Adaptive genetic differentiation

Loci putatively under selection among populations were investigated with PCAdapt v 4.3.3 (Luu et al., 2017; Privé et al., 2020). PCAdapt was run using the allele frequencies of the 18 pools with a Bonferroni *p*‐value adjustment and false discovery rate (FDR) bounded at .05. Applying a Bayesian framework, PCAdapt determines population structure using *K* z‐scores to fit SNPs to *K* principal components based on Cattell's rule (Cattell, 1966), where SNP‐specific Mahalanobis distances are used to evaluate putatively adaptive loci from the normal z‐score distribution, explained by the *K* factors. We chose PCAdapt because of its ability to run pool‐seq data and its strength in examining a divergence model with hierarchical population structure, while maintaining statistical power and a controlled FDR (Luu et al., 2017). Furthermore, PCAdapt has been shown to have consistent strength for detecting putatively adaptive loci under weak, moderate, and strong selection (Lotterhos & Whitlock, 2015).

A redundancy analysis (RDA) was used to determine the driving habitat and environmental factors (*n* = 14; Table 1) of putative loci under selection. The RDA, a form of multivariate genotype–environment association, was conducted using the package vegan v 2.5‐6 (Oksanen et al., 2019) on pooled allele frequency data from putatively adaptive loci attained from PCAdapt, as we were unable to run the RDA on a larger dataset due to computational restrictions. This RDA was also based on habitat and environmental predictors scaled with the *scale* function in R and filtered to a cutoff of 0.7 with psych package v 1.9.12 to avoid multicollinearity. Significance was computed using F‐statistics for each constrained axis (Legendre et al., 2010) to examine whether specific habitat and environmental predictors explained PCAdapt‐based, putatively adaptive loci.

## 3 RESULTS

### 3.1 DNA sequencing

For the 18 pools (i.e., 9 populations with 2 pools per population (one for each sex)), raw sequence counts totaled 10,775,432,330 reads, of which unique reads averaged 431,983,622 in each pool (72.2%) with quality scores of 36 (Figure S1). All pools passed base quality scores, and per sequence GC content was normally distributed around a mean of 44%. Mean filtered depth of coverage among the 18 pools was 15.4X with a standard deviation of 1.7 (Figure S2). There were 68,836,296 total SNPs called, of which 10,721,236 were removed via quality and mapping parameters, 25,898,239 SNPs were removed due to global minor allele frequency restrictions, and 3,726,203 were removed as indels. Therefore, 28,490,618 SNPs were retained for downstream analyses, covering 70.9% (1,539,082,007 bp) of the reference genome (Figure S3). The proportion of each chromosome covered across all libraries, apart from scaffolds, averaged 77.2% (Figure S4). These SNPs were further filtered for analyses to 362,493 common SNP variants among the 18 pools.

### 3.2 Neutral genetic diversity and differentiation

Levels of nucleotide diversity did not differ significantly among pools; in fact, the highest levels of nucleotide diversity were seen in lakes with very different propagule pressure (Table 1). Neutral genetic differentiation among populations was negligible: *F*_ST_ was effectively zero, averaging −0.020 (−0.062 to −0.002; Table 2).

**TABLE 2:** Neutral genetic differentiation between introduced brook trout populations *F*_ST_, based on male pools (“M”) and female (“F”) pools (bottom); male–female pool comparisons within each lake are denoted with asterisks

Population (M)	Cobb	McNair	Dog	Helen	Margaret	Temple	Mud	Olive	Ross
Cobb	−0.023*	−0.032	−0.014	−0.016	−0.002	−0.016	−0.015	−0.013	−0.015
McNair		−0.062*	−0.038	−0.038	−0.025	−0.039	−0.037	−0.037	−0.039
Dog			−0.023*	−0.019	−0.008	−0.020	−0.019	−0.018	−0.019
Helen				−0.023*	−0.008	−0.022	−0.020	−0.019	−0.020
Margaret					−0.022*	−0.008	−0.008	−0.012	−0.007
Temple						−0.021*	−0.020	−0.019	−0.021
Mud							−0.024*	−0.019	−0.020
Olive								−0.024*	−0.019
Ross									−0.022*

Population (F)	Cobb	McNair	Dog	Helen	Margaret	Temple	Mud	Olive	Ross
Cobb	−0.023*	−0.033	−0.014	−0.016	−0.004	−0.017	−0.015	−0.015	−0.017
McNair		−0.062*	−0.038	−0.038	−0.026	−0.038	−0.037	−0.037	−0.039
Dog			−0.023*	−0.020	−0.008	−0.017	−0.019	−0.019	−0.018
Helen				−0.024*	−0.010	−0.019	−0.021	−0.020	−0.019
Margaret					−0.022*	−0.007	−0.010	−0.014	−0.008
Temple						−0.021*	−0.017	−0.018	−0.020
Mud							−0.024*	−0.020	−0.019
Olive								−0.024*	−0.019
Ross									−0.022*

### 3.3 Relationships between neutral genetic diversity and propagule pressure or environmental variables

**FIGURE 2:**
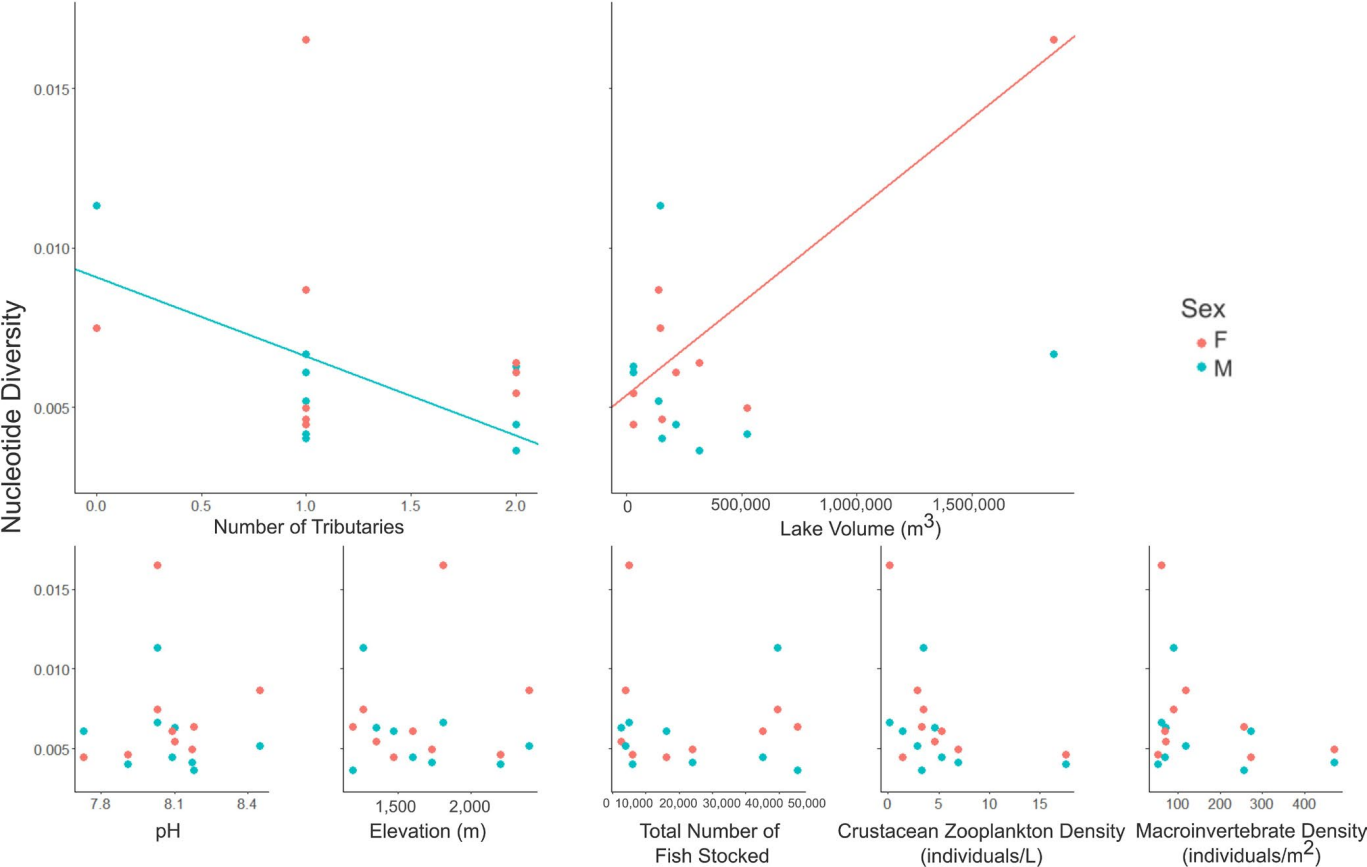
Regressions analyses of nucleotide diversity against tested noncollinear variables. Trends associated with lake volume in female‐based (F) pools and number of tributaries in male‐based pools (M) were statistically significant (trend lines)

Significant beta regressions were negative relationships between nucleotide diversity and lake volume in female populations, and the number of tributaries in male populations (Table S1). With Margaret and Cobb removed (as outliers), the negative trends of lake volume and the number of tributaries remained weakly negative, yet not significant, in females and males, respectively (Figures S5 and S6). Contrary to our hypotheses, populations with larger lake volume (habitat size) had lower nucleotide diversity, while the remaining tested stocking and environmental variables were not drivers of nucleotide diversity in these data (Figure 2). Log transforming the stocking variables associated with propagule pressure did not change the results.

### 3.4 Deleterious mutations within populations

Overall, the results only partially supported our prediction that bottleneck events and a lesser proportion of deleterious mutations would lead to lower neutral genetic variation.

**FIGURE 3:**
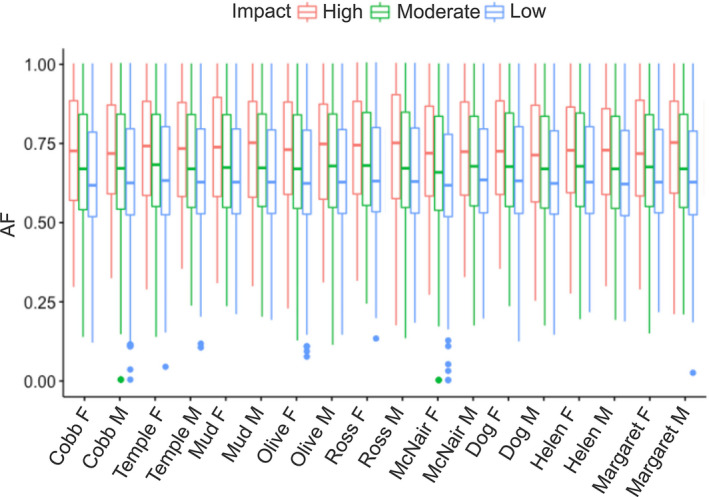
Allele frequency of deleterious loci between populations with three categories of deleterious effect (high, moderate, and low with M = male, F = female)

Putatively highly deleterious mutations (297) had lower allele frequencies than moderate (9,015) and low‐impact (11,030) deleterious mutations, consistent with a role for purifying selection in these populations (Figure 3). *T*‐tests of the mean number of deleterious alleles of all levels (high, moderate, and low) across populations were similar between most populations; however, some populations were significantly different from others (Table S2).

Population‐specific analyses with CMH tests estimated a total of 286 candidate loci that differentiated in allele frequency across all pairwise population comparisons and from 0 to 17 loci per individual pairwise comparison (*n* = 9, scaffold removed; Table 3). Of the 286 loci, 23 either had no exon associated, no results, or an uncharacterized locus. Only four of 286 loci appeared in more than one population; their functions and all population‐specific comparisons are found in Table S3, while no loci with allele frequency differences were associated with putative local adaptation.

**TABLE 3:** SNPs that changed in allele frequency between introduced brook trout populations determined by independent pairwise Cochrane–Mantel–Haenszel analysis (upper), against the number of SNPs in each pairwise analysis (lower) (based on the full, filtered dataset of SNPs including common loci across populations as well as unique ones)

Lake Name	Cobb	Margaret	Olive	Helen	Dog	Ross	Temple	McNair	Mud
Cobb	–	3	8	4	6	3	7	8	2
Margaret	7,659,675	–	10	13	13	10	10	5	0
Olive	7,271,316	7,025,721	–	15	14	10	14	1	1
Helen	7,318,305	7,068,665	6,838,734	–	11	13	10	6	0
Dog	6,531,154	6,450,080	6,308,517	6,311,949	–	11	14	10	1
Ross	7,636,065	7,385,133	7,041,021	7,132,435	6,469,102	–	17	8	0
Temple	6,951,920	6,840,064	6,619,100	6,653,555	6,163,825	6,892,851	–	4	1
McNair	8,246,277	7,728,579	7,347,432	7,456,360	6,692,374	7,820,175	7,152,265	–	2
Mud	7,439,413	7,052,217	6,727,633	6,833,011	6,204,908	7,165,096	6,541,214	7,483,119	–

### 3.5 Adaptive genetic differentiation

**FIGURE 4:**
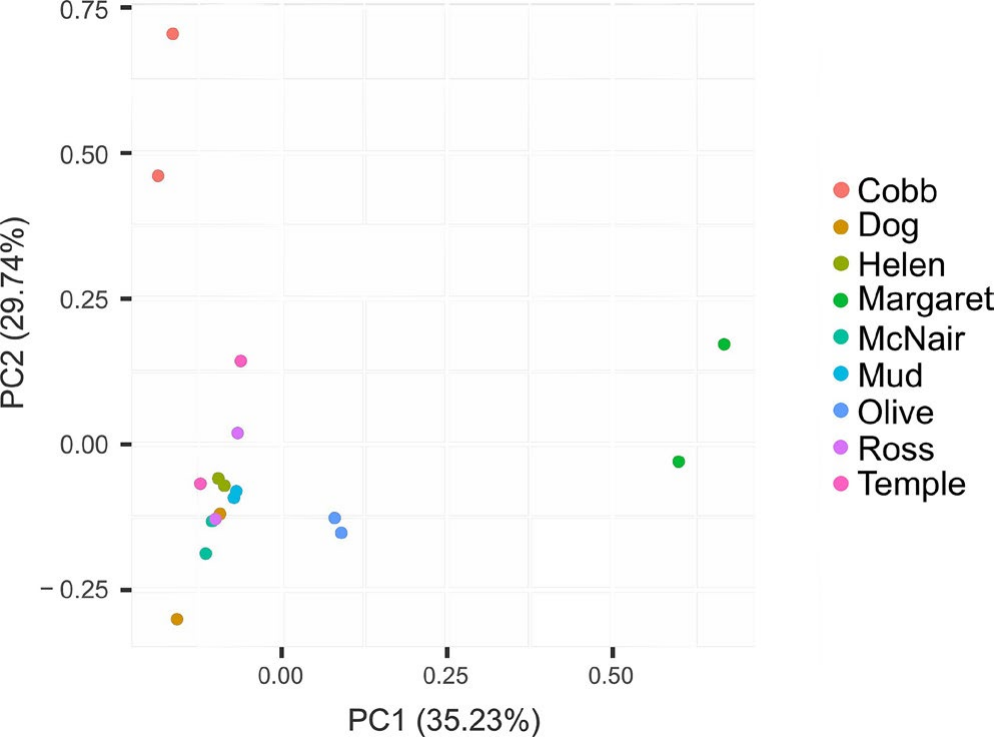
PCAdapt score plot showing the genetic differentiation of introduced brook trout populations

After filtering, PCAdapt identified 2,768 putatively adaptive loci (0.764%) of 362,493 tested SNPs. Male and female pools of each population grouped together in the score plot analysis (Figure 4), while PCAdapt suggested that Cobb and Margaret were somewhat more differentiated than the other seven populations. Contrary to our predictions, the observed putatively adaptive loci under selection were not driven by any tested habitat and environmental variables using RDA (adjusted *R* ^2^ = 2.1%, *p* = .18, with 999 permutations). Re‐analysis of the RDA running VIF analysis <10 to avoid overfitting the model remained non‐significant (adjusted *R* ^2^ = 1.8%, *p* = .11, with 999 permutations), when removing upstream and downstream water discharge, number of tributaries, surface area, depth, temperature variance, and number of discernable spawning sites.

## 4 DISCUSSION

Species introduction success to novel environments is thought to be directly linked to the product of propagule pressure and genomic variation as it facilitates adaptation (Dlugosch et al., 2015; Lee, 2002; Sakai et al., 2001), but empirical investigations using whole‐genome resolution are rare (Dennenmoser et al., 2017; Yoshida et al., 2016). Our pool‐seq study on isolated brook trout populations introduced from a common source into alpine lakes on average ~19 generations ago suggests that wide variation (20‐fold differences) in propagule pressure does not result in proportional standing genetic variation among introduced populations. Nor did we find that wide habitat variation in introduced environments leads to proportional variation in neutral or adaptive variation. First, we found little support for a role of abiotic and biotic environmental variables in affecting neutral genetic diversity among populations despite over 100‐fold variability in these variables (Table 1). Second, when examining putative adaptive loci, there were very low levels of largely population‐specific adaptive differentiation that were seemingly independent from the environmental variables measured (Bolnick et al., 2018).

As founder effects are considered impediments to introduction success, adequate propagule pressure and robust source populations may be adequate to support colonization through increased neutral genetic diversity (Allendorf & Lundquist, 2003; Ellstrand & Elam, 1993; Lavergne & Molofsky, 2007). Despite a range of propagule pressure (one introduction event and as little as 2,500 individuals to 22 such events and up to 55,500 individuals), the introduction effort was enough to result in successful colonization of all study populations and might also help to explain the similar levels of standing genetic variation observed among populations. However, we acknowledge that our study only contains successful colonizations and that unavailable hatchery broodstock genetic diversity metrics may be responsible for any genetic bottlenecks during introduction.

Contrary to previous work across different species and taxa (Bernos & Fraser, 2016; Bert et al., 2007; Briscoe Runquist et al., 2020; Lachmuth et al., 2010; Narum et al., 2017; Schindler & Parker, 2002), we did not definitively find that neutral genetic diversity was positively associated with propagule pressure, habitat size (lake volume), or biotic factors such as prey availability. Furthermore, our study's populations were closed to gene flow effects and immigration from non‐sampled introduced populations, or from the hatchery source. Instead, nucleotide diversity was negatively associated with lake volume in females, and the number of tributaries in males, while no relationships were detected for the other tested variables. The negative trends associated with lake volume were driven largely by Margaret, as it is both the largest lake and the population with the most nucleotide diversity in female populations. We suspect this negative relationship is driven primarily by weak propagule pressure compared to other lakes (Table 1); when Margaret was removed, the relationship was insignificant, but remained weakly negative (Figure S5). Similarly, the negative association between nucleotide diversity and the number of tributaries for male populations is driven by the lack of visible tributaries around Cobb Lake coupled with the most nucleotide diversity in male populations, and when Cobb was removed, the relationship was insignificant, but remained weakly negative (Figure S6).

There was little indication that low proportions of deleterious mutations were associated with low levels of neutral genetic diversity among populations. Instead, most populations had similar levels of deleterious mutations, despite varying propagule pressures. Highly deleterious alleles were significantly less common across populations than moderate or low deleterious mutations, suggesting that purifying selection may be present and purging deleterious mutations. Weak population structure may be driven by the relatively short duration for population differentiation since introduction and a potential lack of major founder effects, or the greater coverage afforded by the pool‐seq methodology, as individual genotyping has a greater association with ascertainment bias and selection of highly polymorphic SNPs (Gaughran et al., 2018; Kurland et al., 2019; Malomane et al., 2018).

Despite previous works showing rapid adaptive evolution in 1–14 generations across different taxa (Hendry et al., 2000; Laurentino et al., 2020; Metz et al., 2020), our genome‐wide analysis of 2,768 putative adaptive loci suggested only very low levels of adaptive differentiation between populations after a mean of ~19 generations postintroduction. More putatively adaptive loci distinguished Cobb and Margaret, perhaps because of a unique food source of amphipods in Cobb and interspecific competition with Westslope Cutthroat trout, lower propagule pressure, and greater habitat/spawning availability in Margaret; all of which are factors known to create genetic variability (Collins, 2011; Martin et al., 1988; Osmond & de Mazancourt, 2013). The pattern of Cobb and Margaret appearing most differentiated in PCAdapt analyses also mirrored their more divergent relationship from other populations in the PCAdapt score plot (Figure 4), implying these two lakes may exhibit greater adaptive differentiation. However, putatively adaptive loci in the RDA did not relate to the abiotic and biotic environmental variables tested and may not have been relevant as they did not correspond to candidate loci with differentiated allele frequencies from the pairwise‐based CMH tests. Of the 28,490,618 SNPs used in CMH testing, there were only a combined total of 286 putative candidate loci across all comparisons with CMH tests, representing 0.001%, none of which were associated with putative local adaptation. Of these loci, a mere four duplicate candidate genes suggest a primary role for nonparallel adaptive evolution, without a link to measured environmental variables or neutral forces. However, gene ontology searches confirmed that although different at a molecular level, the observed candidate loci had similar functions (Table S4). The discontinuity between CMH, PCAdapt, and RDA tests may suggest that (i) candidate genes highlighted by the CMH tests have a polygenic element associated with important biological processes acted on by selection, and/or that (ii) some detected putatively adaptive loci are associated with additional, untested environmental variables or biological processes. In addition, the annotation to the charr genome may have further excluded important genetic components despite strong resolution.

Overall, several biological explanations may explain the low and inconsistent level of adaptive population differentiation. First, although the alpine lake habitats in this study have distinguishing features from the native range of brook trout that should foster adaptive differentiation (Table 1; Beaulieu et al., 2021; Bernos & Fraser, 2016; Harbicht et al., 2014; Hecht et al., 2015; Krueger & May, 1991; Power, 1980; Rieman & Allendorf, 2001), perhaps the species may be preadapted to conditions in alpine lake environments (i.e., freezing temperatures, high elevation). Second, ~50 years since introduction (average ~19 generations) may not be enough time to generate stronger adaptive differentiation, though there is some evidence that this can happen in other invasive species across taxa (e.g., Ghalambor et al., 2015; Hendry et al., 2000; Metz et al., 2020). Third, salmonids exhibit phenotypic plasticity and brook trout in particular are effective colonizers of small headwater stream habitats (Hutchings, 1996; Oomen & Hutchings, 2015; Spens et al., 2007; Wood et al., 2015; Wood & Fraser, 2015; Yates et al., 2019). Plasticity‐mediated population persistence is predicted to buffer against adaptive evolution in new environments (Morris et al., 2014), while the direction of plasticity is generally opposite to the direction of adaptive evolution (Ghalambor et al., 2015). These evolutionary processes may be influencing salmonid colonizations in alpine environments without requiring a process of adaptive differentiation. Fourth, as the study lakes all occur in a similar geological area and alpine environments, the environmental contrasts between them might still be too similar for the environment to maintain adaptive divergence or, alternatively, other adaptive processes are at play (e.g., stabilizing selection). Finally, nuances in population histories from stocking events and subsequent establishment may generate population‐specific idiosyncrasies in neutral and adaptive diversity, consistent with predictions for the consequences of phenotypic plasticity to novel environments.

## 5 CONCLUSIONS

Our genome‐wide analysis using pool‐seq facilitated greater resolution for examining the roles of genetic and environmental factors in colonization of introduced species. Understanding the underlying factors that contribute to successful species colonization is crucial for applications in conservation, mitigating effects on endangered species, and population maintenance (Adams et al., 2000; Higgins & Zanden, 2010; Lodge, 1993). In our study, wide ranges in both environmental and propagule pressure did not lead to significant genetic variation among populations. Moreover, population differentiation and signals of local adaptation were not stronger in conditions expected to promote them. Our work suggests that propagule pressure and environmental predictors of neutral genetic diversity are not mutually exclusive and should be considered together, as, in this study, support through one aspect of these variables may have been responsible for colonization success despite events reducing genetic variation. Our work adds to a growing literature supporting proactive/preventative approaches to invasive species management rather than reactive approaches, as even weak propagule pressure of an effective colonizer can quickly lead to uncontrolled invasion (Kratzer et al., 2020; Leung et al., 2002; Rockwell‐Postel et al., 2020). To better understand the neutral and adaptive differentiation of introduced species, we encourage future analyses to use whole‐genome approaches across a greater range of sample sites that include populations with a large range of times since introduction to accumulate adaptive differentiation.

## CONFLICT OF INTEREST

We declare no conflicts of interest.

## AUTHOR CONTRIBUTIONS

**Brent E. Brookes:** Conceptualization (equal); data curation (lead); formal analysis (lead); funding acquisition (supporting); investigation (lead); methodology (lead); writing – original draft (lead); writing – review and editing (equal). **Hyung‐Bae Jeon:** Data curation (supporting); formal analysis (supporting); methodology (supporting); writing – original draft (supporting); writing – review and editing (equal). **Alison M. Derry:** Conceptualization (equal); data curation (supporting); funding acquisition (equal); writing – review and editing (equal). **John R. Post:** Conceptualization (equal); funding acquisition (equal); writing – review and editing (equal). **Sean Rogers:** Conceptualization (equal); funding acquisition (equal); writing – review and editing (equal). **Shelley Humphries:** Data curation (supporting); resources (supporting). **Dylan J. Fraser:** Conceptualization (equal); data curation (supporting); formal analysis (supporting); funding acquisition (equal); investigation (supporting); methodology (supporting); project administration (lead); resources (lead); supervision (lead); writing – original draft (supporting); writing – review and editing (equal).

## Supporting information

Appendix S1

Appendix S2

## Data Availability

Data are available in Dryad (https://doi.org/10.5061/dryad.np5hqbzvd).
