# Supplementary material for: Neutral and adaptive drivers of genomic change in introduced brook trout (Salvelinus fontinalis) populations revealed by pooled sequencing
Source: Ecol Evol. 2022 Feb 7;12(2):e8584. doi: 10.1002/ece3.8584 (PMC8820109; doi:10.1002/ece3.8584)
Supplement: Supplementary file 1 — Appendix S1 [file ECE3-12-e8584-s002.docx]

**Appendix A: Supplemental Tables and Figures**

***Suppl. Table 1*.** All beta-regression summaries for variable terms.

| **Terms** | **Estimate** | **Std. Error** | **z value** | **p value** | **Pseudo R-squared** |
| --- | --- | --- | --- | --- | --- |
| Nd ~ Elevation F | 0.0612 | 0.1329 | 0.461 | 0.645 | 0.02808 |
| Nd ~ Elevation M | -0.09827 | 0.11368 | -0.864 | 0.387 | 0.09012 |
| Nd ~ Lake volume F | 0.33281 | 0.05477 | 6.076 | 1.23e^-9^ | 0.654 |
| Nd ~ Lake volume M | 0.03051 | 0.11111 | 0.275 | 0.784 | 0.007018 |
| Nd ~ pH F | 0.1147 | 0.1301 | 0.882 | 0.378 | 0.1081 |
| Nd ~ pH M | -0.06721 | 0.11313 | -0.594 | 0.552 | 0.04427 |
| Nd ~ Total number of fish stocked F | -0.05052 | 0.13706 | -0.369 | 0.712 | 0.01612 |
| Nd ~ Total number of fish stocked M | 0.003058 | 0.114757 | 0.027 | 0.979 | 8.88e^-5^ |
| Nd ~ Number of tributaries F | -0.07107 | 0.13363 | -0.532 | 0.595 | 0.03799 |
| Nd ~ Number of tributaries M | -0.25871 | 0.08480 | -3.051 | 0.00228 | 0.3999 |
| Nd ~ Zooplankton density F | -0.2428 | 0.1451 | -1.673 | 0.0943 | 0.2733 |
| Nd ~ Zooplankton density M | -0.1474 | 0.1192 | -1.237 | 0.216 | 0.186 |
| Nd ~ Macroinvertebrate density F | -0.1469 | 0.1398 | -1.051 | 0.293 | 0.155 |
| Nd ~ Macroinvertebrate density M | -0.1125 | 0.1164 | -0.966 | 0.334 | 0.1256 |


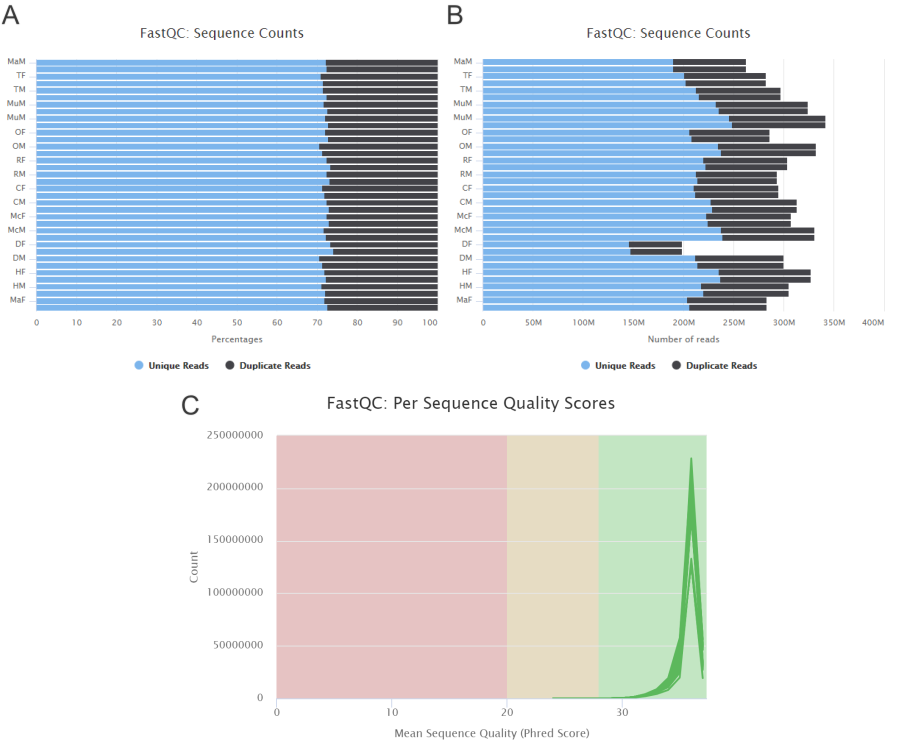


***Suppl. Figure 1*.** FastQC sequence counts visualized as A) percentages and B) number of reads, differentiated between unique and duplicate reads, and C) mean per sequence quality scores. Plots created with MultiQC v 1.7 (Ewels et al., 2016).


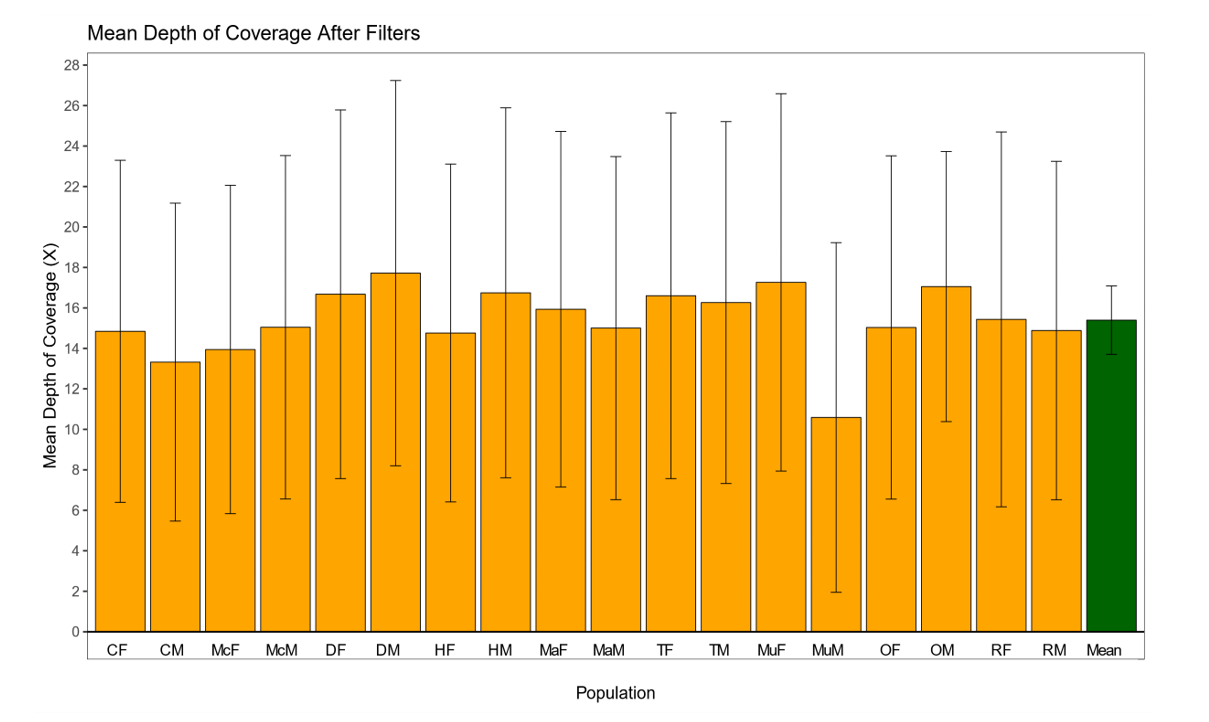


***Suppl. Figure 2*.** Mean depth of coverage across all populations calculated with PPstats in PoolParty v 0.8 (Micheletti & Narum, 2018).


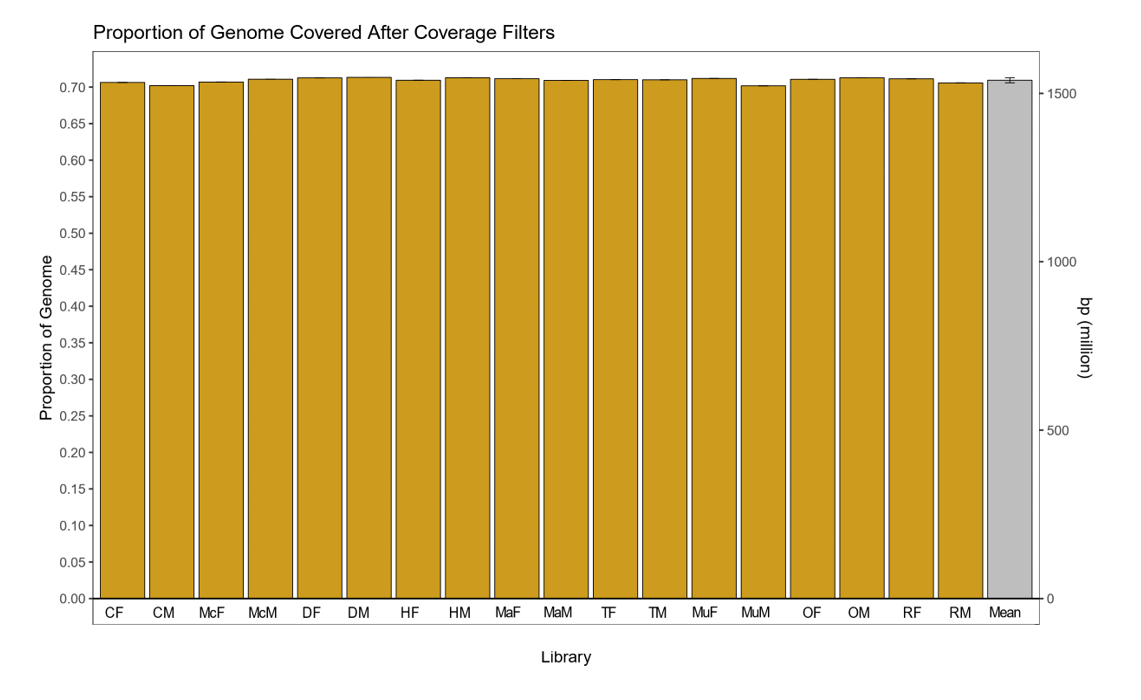


***Suppl. Figure 3.*** Proportion of the genome covered after coverage filters.


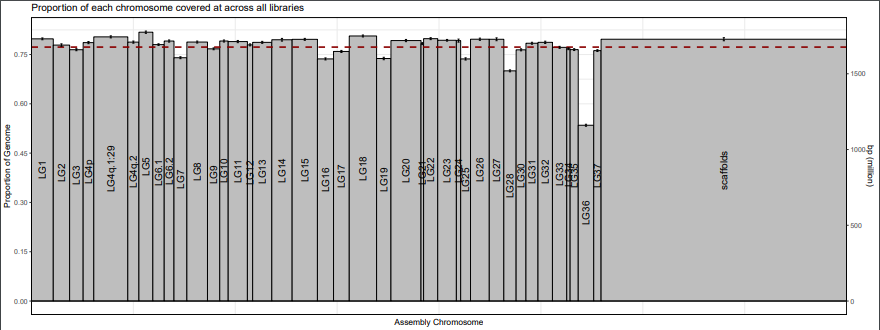


***Suppl. Figure 4*.** Proportion of each chromosome covered by all libraries.

**
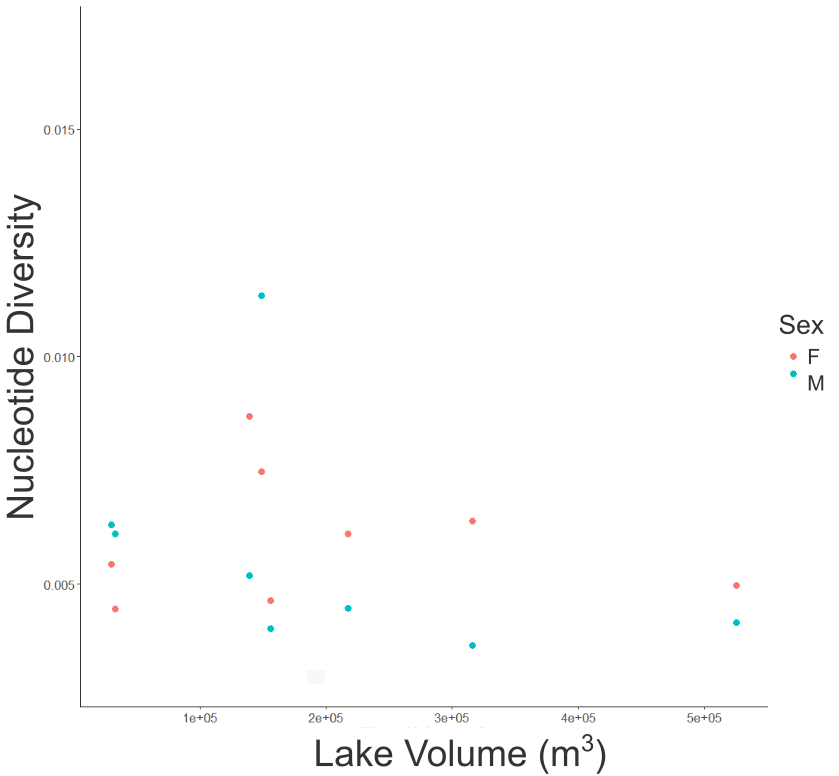
**

***Suppl. Figure 5.*** Nucleotide diversity correlations with lake volume for female (F) and male (M) populations corrected without Lake Margaret.

**
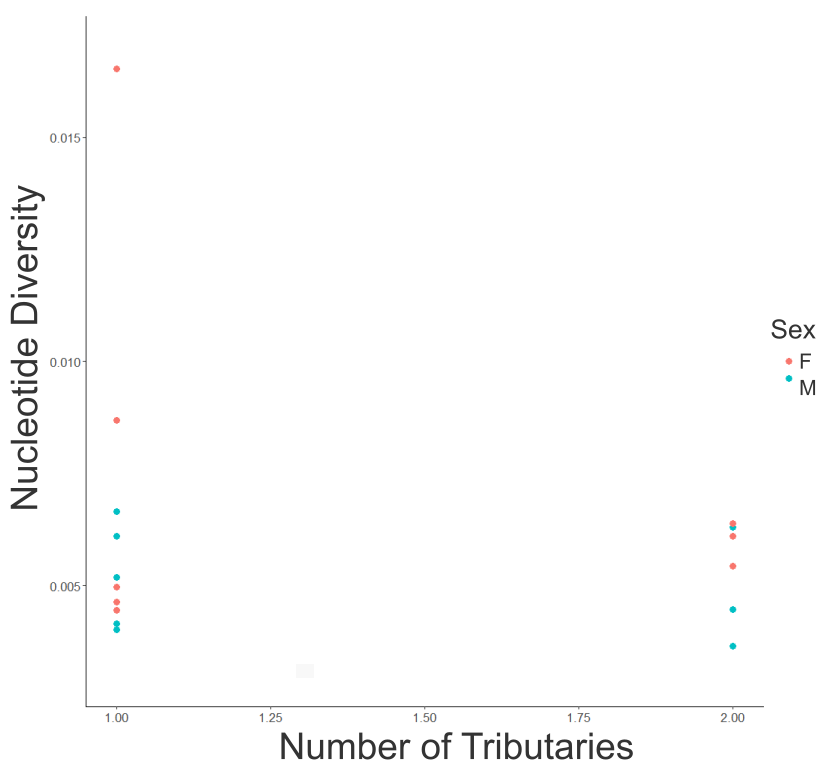
**

***Suppl. Figure 6.*** Nucleotide diversity correlations with the number of tributaries for populations corrected without Cobb Lake.

***Suppl. Table 2.*** Pairwise matrix showing significant differences (* = p < 0.05) in deleterious alleles across populations.

| **Population** | **Cobb** | **McNair** | **Dog** | **Helen** | **Margaret** | **Temple** | **Mud** | **Olive** | **Ross** |
| --- | --- | --- | --- | --- | --- | --- | --- | --- | --- |
| **Cobb** | - | - | - | - | * | * | * | - | * |
| **McNair** |  | - | - | - | * | * | * | - | * |
| **Dog** |  |  | - | - | - | - | - | - | - |
| **Helen** |  |  |  | - | - | * | - | - | * |
| **Margaret** |  |  |  |  | - | - | - | - | - |
| **Temple** |  |  |  |  |  | - | - | - | - |
| **Mud** |  |  |  |  |  |  | - | - | - |
| **Olive** |  |  |  |  |  |  |  | - | * |
| **Ross** |  |  |  |  |  |  |  |  | - |

***Suppl. Table 3.*** Individual lake comparisons through Cochran Mantel Haenszel analysis, with BLAST function, gene ontology category, gene ontology function and the species related to the gene ontology results. Lakes are abbreviated C = Cobb, D = Dog, H = Helen, Mc = McNair, Mu = Mud, Ma = Margaret, O = Olive, R = Ross, and T = Temple. The four of 286 outlier loci appearing in more than one population were: 1) a peptidyl-tRNA hydrolase 1 homolog, categorized as a molecular function, associated with aminoacyl-tRNA hydrolase activity was present in Temple-Helen and Olive-Helen comparisons; 2) a spectrin beta chain, erythrocytic, categorized as a molecular function, associated with actin binding was present in Margaret-McNair and Dog- McNair comparisons; 3) a SPT16 homolog, facilitates chromatin remodeling subunit, categorized as a cellular component, associated with the FACT complex was present in Temple-Olive and Ross-Olive; and 4) an oligosaccharyltransferase complex subunit, categorized as a biological process, associated with protein glycosylation was present in Temple-Olive and Mud-Olive comparisons.

| Lakes | Chromosome | Base Pair | SNP | P value | BLAST Function | Gene Ontology Category | Gene Ontology Function | | | Species | |
| --- | --- | --- | --- | --- | --- | --- | --- | --- | --- | --- | --- |
| TvMc | LG6.1 | 18227277 | snp7 | 0.02242028 | myelin basic protein-like (LOC111965264), transcript variant X1, mRNA | molecular function | structural constituent of myelin sheath | | | *Austrofundulus limnaeus* | |
| TvMc | LG8 | 41620402 | snp11 | 0.04147309 | putative sodium-coupled neutral amino acid transporter 10 (LOC111967963), mRNA | cellular component | membrane | | | *Liparis tanakae* | |
| TvMc | LG15 | 29900965 | snp15 | 0.01294434 | transport and golgi organization 2 homolog (tango2), transcript variant X3, mRNA | cellular component | Golgi apparatus | | | *Equus caballus* | |
| TvMc | LG37 | 13533282 | snp36 | 0.000146782 | long-chain-fatty-acid--CoA ligase ACSBG2 (LOC111972157), transcript variant X2, mRNA | molecular function | ligase activity | | | *Phytophthora nicotianae* | |
| CvMc | LG4q.1:29 | 60884645 | snp5 | 0.01817296 | SNF-related serine/threonine-protein kinase-like (LOC111962281), mRNA | molecular function | protein kinase activity | | | *Scleropages formosus* | |
| CvMc | LG15 | 34473273 | snp6 | 0.02917038 | inactive carboxypeptidase-like protein X2 (LOC111974979), mRNA | molecular function | metallocarboxypeptidase activity | | | *Mus musculus* | |
| CvMc | LG15 | 46322243 | snp7 | 5.05E-06 | homeobox protein orthopedia B-like (LOC111975202), transcript variant X1, mRNA | cellular component | nucleus | | | *Aurelia aurita* | |
| CvMc | LG15 | 64538541 | snp8 | 0.04471552 | DENN domain containing 5A (dennd5a), transcript variant X4, mRNA | cellular component | trans-Golgi network | | | *Aotus nancymaae* | |
| CvMc | LG16 | 10436059 | snp9 | 0.02562949 | protein FAM43A-like (LOC111976060), mRNA | cellular component | mitochondrion | | | *Ectocarpus siliculosus* | |
| CvMc | LG28 | 19394862 | snp17 | 0.01951748 | protein phosphatase 4 regulatory subunit 4 (ppp4r4), transcript variant X1, mRNA | cellular component | cytosol | | | *Gorilla gorilla gorilla* | |
| CvMc | LG32 | 20614849 | snp21 | 0.006108301 | G-protein coupled receptor 54 (LOC111956315), mRNA | molecular function | G protein-coupled receptor activity | | | *Ophiophagus hannah* | |
| CvMc | LG36 | 40398713 | snp24 | 0.006956964 | dedicator of cytokinesis protein 9-like (LOC111959519), mRNA | molecular function | guanyl-nucleotide exchange factor activity | | | *Scleropages formosus* | |
| TvH | LG5 | 14518627 | snp8 | 9.72E-05 | collagen alpha-2(XI) chain-like (LOC111963946), mRNA | cellular component | collagen trimer | | | *Penaeus vannamei* | |
| TvH | LG6.1 | 22847223 | snp10 | 0.001711872 | transmembrane emp24 domain-containing protein 11 (LOC111965330), transcript variant X1, mRNA | cellular component | Golgi apparatus | | | *Mus musculus* | |
| TvH | LG6.2 | 16908776 | snp12 | 0.000167943 | multidrug resistance-associated protein 5 (LOC111965891), mRNA | molecular function | organic anion transmembrane transporter activity | | | *Phaethon lepturus* | |
| TvH | LG8 | 32338519 | snp13 | 0.02055054 | glutamate receptor ionotropic, delta-1-like (LOC111967743), mRNA | molecular function | ionotropic glutamate receptor activity | | | *Ictalurus punctatus* | |
| TvH | LG15 | 14478967 | snp17 | 0.001781122 | peptidyl-tRNA hydrolase 1 homolog (ptrh1), transcript variant X4, mRNA | molecular function | aminoacyl-tRNA hydrolase activity | | | *Paramormyrops kingsleyae* | |
| TvH | LG22 | 5565375 | snp22 | 9.78E-06 | B-cell lymphoma/leukemia 11A-like (LOC111949579), transcript variant X1, mRNA | molecular function | nucleic acid binding | | | *Austrofundulus limnaeus* | |
| TvH | LG23 | 33592297 | snp25 | 5.48E-06 | seizure protein 6-like (LOC111950797), transcript variant X1, mRNA | cellular component | membrane | | | *Macaca fascicularis* | |
| TvH | LG23 | 43182717 | snp26 | 0.03394847 | CCR4-NOT transcription complex subunit 6 (LOC111950253), mRNA | cellular component | membrane | | | *Trachymyrmex cornetzi* | |
| TvH | LG28 | 14531622 | snp31 | 0.000900496 | human immunodeficiency virus type I enhancer binding protein 2 (hivep2), mRNA | molecular function | nucleic acid binding | | | *Cebus capucinus imitator* | |
| TvH | LG28 | 31400198 | snp33 | 0.003921092 | WAS protein family member 1 (wasf1), mRNA | biological process | actin cytoskeleton organization | | | *Lates calcarifer* | |
| MavMc | LG1 | 8555253 | snp2 | 0.01917777 | mitochondrial import receptor subunit TOM20 homolog (LOC111960523), mRNA | cellular component | mitochondrial outer membrane translocase complex | | | *Austrofundulus limnaeus* | |
| MavMc | LG7 | 10773859 | snp13 | 0.000381234 | tumor necrosis factor receptor superfamily member 6B-like (LOC111966075), mRNA | biological process | signal transduction | | | *Callorhinchus milii* | |
| MavMc | LG8 | 46763424 | snp15 | 7.61E-06 | S1 RNA binding domain 1 (srbd1), transcript variant X1, mRNA | cellular component | membrane | | | *Xenopus tropicalis* | |
| MavMc | LG9 | 3762720 | snp18 | 0.00767866 | spectrin beta chain, erythrocytic (LOC111968456), transcript variant X2, mRNA | molecular function | actin binding | | | *Tinamus guttatus* | |
| MavMc | LG10 | 2902731 | snp19 | 0.02638717 | protein FMC1 homolog (LOC111980476), mRNA | cellular component | mitochondrion | | | *Xenopus tropicalis* | |
| CvMu | LG9 | 15711057 | snp1 | 0.00740331 | CDK-activating kinase assembly factor MAT1-like (LOC111968663), transcript variant X1, mRNA | cellular component | transcription factor TFIIH holo complex | | | *Leptotrombidium deliense* | |
| CvMu | LG11 | 31603784 | snp3 | 0.006384625 | SPRY domain-containing protein 3 (LOC111970186), transcript variant X3, mRNA | molecular function | Dna binding | | | *Clunio marinus* | |
| MavH | LG1 | 36458623 | snp2 | 0.00767866 | 6-phosphofructo-2-kinase/fructose-2,6-bisphosphatase 4 (LOC111966555), transcript variant X2, mRNA | molecular function | 6-phosphofructo-2-kinase activity | | | *Macaca fascicularis* | |
| MavH | LG4p | 19447940 | snp6 | 0.02399268 | regulation of nuclear pre-mRNA domain-containing protein 2 (LOC111960658), transcript variant X1, misc_RNA | molecular function | aminoacyl-tRNA ligase activity | | | *Chelonia mydas* | |
| MavH | LG4p | 22774374 | snp7 | 0.02175842 | LIM/homeobox protein Lhx1-like (LOC111960709), transcript variant X1, mRNA | molecular function | sequence-specific DNA binding | | | *Austrofundulus limnaeus* | |
| MavH | LG6.2 | 16763358 | snp12 | 0.000355827 | Bruton tyrosine kinase (btk), transcript variant X4, mRNA | biological process | negative regulation of intrinsic apoptotic signaling pathway | | | *Xenopus tropicalis* | |
| MavH | LG7 | 14675173 | snp14 | 0.006384625 | rho GTPase-activating protein 39 (LOC111966455), mRNA | biological process | signal transduction | | | *Lygus hesperus* | |
| MavH | LG13 | 19370915 | snp20 | 0.04147309 | protein-glutamine gamma-glutamyltransferase K (LOC111972247), mRNA | molecular function | transferase activity | | | *Mizuhopecten yessoensis* | |
| MavH | LG17 | 37935612 | snp25 | 0.03163034 | ATP-sensitive inward rectifier potassium channel 12-like (LOC111976506), mRNA | molecular function | voltage-gated ion channel activity | | | *Scleropages formosus* | |
| MavH | LG20 | 70063720 | snp29 | 0.007104126 | interferon alpha-like (LOC111980627), mRNA | molecular function | cytokine receptor binding | | | *Enhydra lutris kenyoni* | |
| MavH | LG25 | 10937222 | snp33 | 0.000712784 | collagen alpha-1(XIII) chain (LOC111951857), transcript variant X26, mRNA | cellular component | collagen trimer | | | *Fukomys damarensis* | |
| MavH | LG27 | 5827389 | snp38 | 0.01402871 | activin receptor type-2B (LOC111953775), mRNA | molecular function | transferase activity | | | *Rattus norvegicus* | |
| MavH | LG27 | 13913073 | snp40 | 1.68E-06 | dnaJ homolog subfamily C member 13 (LOC111953250), transcript variant X8, mRNA | cellular component | endosome | | | *Homo sapiens* | |
| MavH | LG34 | 8953008 | snp47 | 0.03776503 | E3 ubiquitin-protein ligase ZFP91 (LOC111958372), mRNA | molecular function | nucleic acid binding | | | *Phoenicopterus ruber ruber* | |
| MavH | LG36 | 9940106 | snp49 | 0.04335784 | bromodomain adjacent to zinc finger domain 2B (baz2b), transcript variant X16, mRNA | cellular component | host cell nucleus | | | *Poecilia formosa* | |
| CvH | LG3 | 19366919 | snp3 | 0.03907941 | Nance-Horan syndrome protein-like (LOC111956283), mRNA | biological process | actin cytoskeleton organization | | | *Scleropages formosus* | |
| CvH | LG21 | 6029309 | snp10 | 0.01378619 | zinc finger protein 180-like (LOC111982340), transcript variant X4, mRNA | molecular function | nucleic acid binding | | | *Salmo salar* | |
| CvH | LG23 | 21752291 | snp11 | 0.000104212 | protein FAM114A2 (LOC111950452), transcript variant X1, mRNA | biological process | biological process | | | *Mus musculus* | |
| CvH | LG24 | 5494728 | snp12 | 0.00259454 | metabotropic glutamate receptor 8-like (LOC111951335), mRNA | molecular function | G protein-coupled receptor activity | | | *Limosa lapponica baueri* | |
| CvT | LG6.1 | 28928483 | snp5 | 0.001865465 | platelet-derived growth factor receptor beta (LOC111965401), transcript variant X3, mRNA | molecular function | platelet-derived growth factor beta-receptor activity | | | *Chlorocebus sabaeus* | |
| CvT | LG6.2 | 11612437 | snp6 | 9.23E-05 | elongation of very long chain fatty acids protein 6 (LOC111965827), mRNA | cellular component | endoplasmic reticulum membrane | | | *Larimichthys crocea* | |
| CvT | LG8 | 22898258 | snp8 | 7.43E-05 | short transient receptor potential channel 3-like (LOC111967605), mRNA | molecular function | calcium channel activity | | | *Brachionus plicatilis* | |
| CvT | LG8 | 35963603 | snp10 | 0.001686152 | carbonic anhydrase-related protein 10 (LOC111967825), transcript variant X1, mRNA | molecular function | zinc ion binding | | | *Camelus dromedarius* | |
| CvT | LG11 | 21851796 | snp14 | 0.000635082 | heterochromatin protein 1-binding protein 3 (LOC111969980), transcript variant X1, mRNA | cellular component | nucleosome | | | *Heterocephalus glaber* | |
| CvT | LG13 | 34965927 | snp15 | 2.12E-05 | low-density lipoprotein receptor-related protein 8-like (LOC111971375), transcript variant X2, mRNA | molecular function | calcium ion binding | | | *Ictalurus punctatus* | |
| CvT | LG20 | 31257947 | snp20 | 0.000217396 | family with sequence similarity 168 member A (fam168a), transcript variant X2, mRNA | biological process | positive regulation of base-excision repair | | | *Ursus maritimus* | |
| MavT | LG1 | 20615374 | snp3 | 0.01917777 | disks large-associated protein 4-like (LOC111962313), mRNA | biological process | signaling | | | *Physeter macrocephalus* | |
| MavT | LG2 | 19816607 | snp6 | 0.02153945 | rho-related GTP-binding protein RhoE (LOC111976449), mRNA | molecular function | GTP binding | | | *Nipponia nippon* | |
| MavT | LG9 | 9097812 | snp17 | 0.04257932 | zinc finger MYM-type protein 4 (LOC111968537), mRNA | molecular function | zinc ion binding | | | *Eurypyga helias* | |
| MavT | LG11 | 14766062 | snp18 | 0.00767866 | CaM kinase like vesicle associated (camkv), mRNA | molecular function | zinc ion binding | | | *Xenopus tropicalis* | |
| MavT | LG12 | 11887432 | snp23 | 0.000614582 | neural cell adhesion molecule 2 (ncam2), mRNA | biological process | cell adhesion | | | *Nothobranchius furzeri* | |
| MavT | LG13 | 18242153 | snp24 | 0.007956303 | neuroligin-1-like (LOC111972231), mRNA | cellular component | membrane | | | *Tropilaelaps mercedesae* | |
| MavT | LG15 | 48555482 | snp32 | 0.003123204 | serine/threonine-protein kinase BRSK2 (LOC111974396), mRNA | molecular function | protein kinase activity | | | *Lygus hesperus* | |
| MavT | LG17 | 28566718 | snp35 | 0.02562949 | activin receptor type-1B-like (LOC111976988), transcript variant X2, mRNA | biological process | transmembrane receptor protein serine/threonine kinase signaling pathway | | | *Xenopus* | |
| MavT | LG23 | 14747480 | snp43 | 0.003032185 | roundabout homolog 3 (LOC111950617), transcript variant X3, mRNA | cellular component | plasma membrane | | | *Nothobranchius rachovii* | |
| MavT | LG33 | 14386258 | snp52 | 0.01263083 | frizzled-10-B (LOC111957788), mRNA | biological process | Wnt signaling pathway | | | *Ascaris suum* | |
| CvR | LG4p | 25157850 | snp3 | 0.006108301 | chloride channel protein 2-like (LOC111960749), mRNA | cellular component | membrane | | | *Salmo salar* | |
| CvR | LG25 | 20912172 | snp15 | 0.02175842 | neuregulin 3 (nrg3), transcript variant X1, mRNA | molecular function | signaling receptor binding | | | *Macaca nemestrina* | |
| CvR | LG36 | 8603537 | snp20 | 0.02803754 | double-stranded RNA-specific editase 1 (LOC111959749), transcript variant X4, mRNA | molecular function | adenosine deaminase activity | | | *Lygus hesperus* | |
| MavR | LG3 | 26942053 | snp5 | 0.003217101 | arf-GAP with coiled-coil, ANK repeat and PH domain-containing protein 1-like (LOC111953962), mRNA | molecular function | metal ion binding | | | *Scleropages formosus* | |
| MavR | LG4q.1:29 | 81602808 | snp9 | 0.000678933 | cat eye syndrome critical region protein 2 (LOC111962616), transcript variant X1, mRNA | biological process | ATP-dependent chromatin remodeling | | | *Trichinella patagoniensis* | |
| MavR | LG11 | 37588105 | snp16 | 0.000175444 | U3 small nucleolar RNA-interacting protein 2 (LOC111970321), mRNA | biological process | rRNA processing | | | *Malassezia restricta CBS 7877* | |
| MavR | LG15 | 23946954 | snp17 | 6.15E-05 | arginine/serine-rich coiled-coil protein 2 (LOC111974230), mRNA | biological process | biological process | | | *Mus musculus* | |
| MavR | LG17 | 33103754 | snp24 | 2.57E-07 | multiple epidermal growth factor-like domains protein 6 (LOC111976271), mRNA | molecular function | calcium ion binding | | | *Trichinella pseudospiralis* | |
| MavR | LG18 | 25704590 | snp26 | 0.000821241 | peroxisome proliferator-activated receptor gamma coactivator-related protein 1 (LOC111978040), mRNA | molecular function | transcription coregulator activity | | | *Mus musculus* | |
| MavR | LG31 | 5864471 | snp34 | 0.02175842 | choline transporter-like protein 4 (LOC111955469), mRNA | cellular component | membrane | | | *Operophtera brumata* | |
| MavR | LG33 | 1089947 | snp37 | 0.001111816 | protein fem-1 homolog C (LOC111958058), mRNA | cellular component | nucleoplasm | | | *Physeter macrocephalus* | |
| MavR | LG33 | 29830872 | snp39 | 0.000295767 | serine/threonine-protein kinase A-Raf (LOC111957871), transcript variant X4, mRNA | molecular function | protein kinase activity | | | *Termitomyces sp. J132* | |
| MavR | LG36 | 19606343 | snp40 | 0.004247934 | rho guanine nucleotide exchange factor 7-like | molecular function | guanyl-nucleotide exchange factor activity | | | *Scleropages formosus* | |
| CvD | LG4q.1:29 | 81001122 | snp8 | 0.002057949 | transmembrane and TPR repeat-containing protein 2-like (LOC111962607), transcript variant X1, mRNA | cellular component | integral component of membrane | | | *Scleropages formosus* | |
| CvD | LG8 | 16993680 | snp10 | 0.005706209 | Bardet-Biedl syndrome 12 (bbs12), mRNA | biological process | convergent extension involved in gastrulation | | | *Fundulus heteroclitus* | |
| CvD | LG15 | 48376592 | snp16 | 9.78E-06 | Ras association domain family member 7 (rassf7), transcript variant X1, mRNA | biological process | regulation of microtubule cytoskeleton organization | | | *Ursus maritimus* | |
| CvD | LG16 | 145476 | snp17 | 0.000254794 | vitellogenin-like (LOC111975854), mRNA | molecular function | lipid transporter activity | | | *Agrilus planipennis* | |
| CvD | LG19 | 35153409 | snp19 | 4.91E-06 | CMRF35-like molecule 4 (LOC111979088), mRNA | cellular component | membrane | | | *Labeo rohita* | |
| CvD | LG20 | 69342944 | snp20 | 1.42E-05 | protein FAM171A2 (LOC111980925), mRNA | cellular component | integral component of membrane | | | *Ursus maritimus* | |
| MavD | LG1 | 3654619 | snp1 | 0.0175152600 | keratin, type I cytoskeletal 13-like (LOC111982613), mRNA | molecular function | structural molecule activity | | | *Callorhinchus milii* | |
| MavD | LG13 | 4510701 | snp24 | 0.0039210920 | troponin I, fast skeletal muscle (LOC111971996), transcript variant X1, mRNA | cellular component | troponin complex | | | *Fundulus heteroclitus* | |
| MavD | LG13 | 1312365 | snp23 | 0.0057062090 | transcription factor SOX-6-like (LOC111971958), mRNA | molecular function | DNA binding | | | *Salmo salar* | |
| MavD | LG14 | 48718398 | snp27 | 0.0425477400 | coagulation factor XIII A chain-like (LOC111973567), mRNA | cellular component | membrane | | | *Chlorella sorokiniana* | |
| MavD | LG18 | 44275061 | snp32 | 0.0006789325 | pro-neuregulin-3, membrane-bound isoform-like (LOC111978446), mRNA | cellular component | integral component of membrane | | | *Austrofundulus limnaeus* | |
| MavD | LG18 | 58843553 | snp34 | 0.0377650300 | vinculin (LOC111978256), transcript variant X1, mRNA | molecular function | actin binding | | | *Scolopendra viridis* | |
| MavD | LG23 | 44538747 | snp38 | 0.0420096200 | junctional adhesion molecule A (LOC111950569), mRNA | biological process | cell adhesion | | | *Sparus aurata* | |
| MavD | LG26 | 37452614 | snp39 | 0.0054464470 | transmembrane and TPR repeat-containing protein 2-like (LOC111952765), transcript variant X1, mRNA | cellular component | integral component of membrane | | | *Scleropages formosus* | |
| MavD | LG26 | 41144937 | snp40 | 0.0398734300 | mitochondrial inner membrane protease subunit 2 (LOC111952461), mRNA | biological process | proteolysis | | | *Kwoniella dejecticola CBS 10117* | |
| MavD | LG27 | 16829360 | snp42 | 0.0000471113 | rab effector MyRIP (LOC111953573), mRNA | molecular function | metal ion binding | | | *Fukomys damarensis* | |
| MavD | LG28 | 28727331 | snp48 | 0.0052619750 | pre-mRNA-splicing regulator WTAP (LOC111954449), transcript variant X5, mRNA | biological process | regulation of alternative mRNA splicing, via spliceosome | | | *Opisthocomus hoazin* | |
| MavD | LG3 | 30073875 | snp6 | 0.0007596940 |  |  |  | | |  | |
| MavD | LG3 | 31827266 | snp7 | 0.0267688100 | homeobox protein Hmx-like (LOC111981440), mRNA | cellular component | nucleus | | | *Clunio marinus* | |
| MavD | LG5 | 22106851 | snp15 | 0.0012601530 | uncharacterized LOC111964057 (LOC111964057), transcript variant X2, mRNA | | | | | | |
| MavD | LG5 | 13076882 | snp14 | 0.0050693100 | erbin (LOC111963937), transcript variant X1, mRNA | molecular function | ErbB-2 class receptor binding | | | *Felis catus* | |
| CvO | LG3 | 29380169 | snp2 | 0.03698202 | Kv channel-interacting protein 4-like (LOC111954260), transcript variant X2, mRNA | molecular function | calcium ion binding | | | *Scleropages formosus* | |
| CvO | LG4q.2 | 26534720 | snp7 | 0.000186381 | coiled-coil domain containing 170 (ccdc170), transcript variant X1, mRNA | cellular component | integral component of membrane | | | *Esox lucius* | |
| CvO | LG9 | 8329517 | snp14 | 0.02188711 | A-kinase anchor protein 6-like (LOC111968513), transcript variant X1, mRNA | molecular  function | kinase activity | | | *Austrofundulus limnaeus* | |
| CvO | LG9 | 30238262 | snp17 | 0.01917777 | arf-GAP with SH3 domain, ANK repeat and PH domain-containing protein 2-like (LOC111968391), mRNA | molecular function | GTPase activator activity | | | *Hirondellea gigas* | |
| CvO | LG14 | 48060865 | snp18 | 0.04227848 | E3 ubiquitin-protein ligase RNF152 (LOC111973382), mRNA | molecular function | ubiquitin protein ligase activity | | | *Homo sapiens* | |
| CvO | LG16 | 1455863 | snp20 | 0.002851459 | tubulin tyrosine ligase like 7 (ttll7), transcript variant X1, mRNA | biological process | cellular protein modification process | | | *Papio anubis* | |
| CvO | LG31 | 9973521 | snp25 | 1.02E-06 | collagen alpha-1(XI) chain-like (LOC111955648), mRNA | cellular component | collagen trimer | | | *Alligator mississippiensis* | |
| CvO | LG33 | 8509449 | snp26 | 0.009117259 | protein FAM222A (LOC111957935), mRNA | biological process | biological process | | | *Mus musculus* | |
| MavO | LG4p | 19029998 | snp2 | 0.00825757 | endothelin-converting enzyme 2-like | biological process | proteolysis | | | *Scleropages formosus* | |
| MavO | LG4q.1:29 | 46734565 | snp6 | 0.02676881 | leucine rich repeat and Ig domain containing 1 (lingo1), mRNA | molecular function | epidermal growth factor receptor binding | | | *Macaca mulatta* | |
| MavO | LG5 | 35326545 | snp8 | 0.009217599 | no exon |  |  | | |  | |
| MavO | LG6.2 | 1950395 | snp9 | 0.006108301 | pinopsin-like | molecular function | G protein-coupled receptor activity | | | *Stylophora pistillata* | |
| MavO | LG6.2 | 3382951 | snp10 | 0.01291537 | G protein-coupled receptor activity (mrpl11), transcript variant X1, mRNA | biological process | RNA processing | | | *Xenopus tropicalis* | |
| MavO | LG6.2 | 18765852 | snp11 | 0.005261975 | extensin | molecular function | structural constituent of cell wall | | | *Trifolium pratense* | |
| MavO | LG7 | 33207539 | snp12 | 0.006534903 | keratin, type II cytoskeletal 8 (LOC111966854), mRNA | molecular function | protein binding | | | *Homo sapiens* | |
| MavO | LG9 | 31657974 | snp18 | 0.04227848 | MAM domain containing glycosylphosphatidylinositol anchor 2 (mdga2), mRNA | cellular component | membrane | | | *Iconisemion striatum* | |
| MavO | LG16 | 8841445 | snp33 | 0.00722728 | paralemmin-1 (LOC111975564), mRNA | biological process | regulation of flower development | | | *Anthurium amnicola* | |
| MavO | LG16 | 40816692 | snp35 | 0.001639909 | uncharacterized LOC111975783 (LOC111975783), ncRNA | | | | | | |
| MavO | LG17 | 34432365 | snp37 | 0.03389485 | mitochondrial carrier homolog 1 (LOC111976240), transcript variant X1, mRNA | cellular component | membrane | | | *Homo sapiens* | |
| MavO | LG20 | 74151134 | snp40 | 0.04013898 | BAI1 associated protein 3 (baiap3), mRNA | biological process | dense core granule maturation | | | *Chlorocebus sabaeus* | |
| MavO | LG32 | 4348632 | snp55 | 0.02487944 | uncharacterized LOC111957222 (LOC111957222), transcript variant X1, mRNA | | | | | | |
| CvMa | LG6.2 | 20299408 | snp5 | 0.003217101 | PH and SEC7 domain-containing protein 2 (LOC111965965), mRNA | molecular function | ARF guanyl-nucleotide exchange factor activity | | | *Tinamus guttatus* | |
| CvMa | LG11 | 6898829 | snp9 | 0.003217101 | protein kinase C and casein kinase substrate in neurons protein 1 (LOC111969716), transcript variant X3, mRNA | molecular function | kinase activity | | | *Trichinella pseudospiralis* | |
| CvMa | LG18 | 38344797 | snp16 | 0.003921092 | protocadherin-15-like (LOC111977924), mRNA | molecular function | calcium ion binding | | | *Salmo salar* | |
| CvMa | LG31 | 30807570 | snp25 | 0.00029764 | succinate dehydrogenase [ubiquinone] flavoprotein subunit, mitochondrial (LOC111955807), mRNA | no results |  | | |  | |
| CvMa | LG36 | 17153082 | snp30 | 0.02796417 | uncharacterized LOC111959851 (LOC111959851), ncRNA | | | | | | |
| DvH | LG2 | 39334720 | snp5 | 0.007708939 | alpha-1,3-mannosyl-glycoprotein 4-beta-N-acetylglucosaminyltransferase A-like (LOC111979746), mRNA | molecular function | transferase activity | | | *Scleropages formosus* | |
| DvH | LG4q.2 | 5198062 | snp7 | 0.04553445 | mucolipin-2-like (LOC111962952), mRNA | molecular function | cation channel activity | | | *Enhydra lutris kenyoni* | |
| DvH | LG14 | 17507156 | snp16 | 0.02917038 | calcipressin-2 (LOC111972542), transcript variant X2, mRNA | molecular function | nucleic acid binding | | | *Dryobates pubescens* | |
| DvH | LG18 | 31006510 | snp24 | 0.002621822 | echinoderm microtubule associated protein like 4 (eml4), mRNA | cellular component | membrane | | | *Nothobranchius furzeri* | |
| DvH | LG18 | 54757101 | snp28 | 8.33E-05 | neuromedin-K receptor-like (LOC111977665), mRNA | cellular component | integral component of membrane | | | *Actinidia chinensis var. chinensis* | |
| DvH | LG20 | 2274725 | snp29 | 0.015025 | histone deacetylase 5-like (LOC111981176), mRNA | molecular function | hydrolase activity | | | *Nelumbo nucifera* | |
| DvH | LG20 | 64290763 | snp31 | 0.04394857 | protein FAM163B-like (LOC111980058), mRNA | cellular component | membrane | | | *Scleropages formosus* | |
| DvH | LG22 | 4205787 | snp35 | 0.02715229 | uncharacterized LOC111949697 (LOC111949697), mRNA | | | | | | |
| DvH | LG26 | 20987803 | snp39 | 0.008020971 | breast cancer anti-estrogen resistance protein 1 (LOC111952497), transcript variant X3, mRNA | biological process | signal transduction | | | *Castor canadensis* | |
| DvH | LG26 | 35585050 | snp41 | 0.04335784 | ankyrin repeat and BTB/POZ domain-containing protein BTBD11-B (LOC111952570), transcript variant X1, mRNA | molecular function | metal ion binding | | | *Human parainfluenza 2 virus* | |
| DvH | LG30 | 18719397 | snp44 | 0.009419916 | zinc finger protein 574 (LOC111954987), transcript variant X3, mRNA | molecular function | nucleic acid binding | | | *Chlorocebus sabaeus* | |
| DvH | LG32 | 13801416 | snp46 | 0.00494694 | alpha-tocopherol transfer protein (LOC111956960), transcript variant X2, mRNA | cellular component | ribosome | | | *Camelus dromedarius* | |
| MuvMc | LG4q.1:29 | 30640310 | snp3 | 0.04227848 | RNA exonuclease 4 (LOC111961803), transcript variant X1, mRNA | cellular component | nuclear speck | | | *Homo sapiens* | |
| MuvMc | LG30 | 6585886 | snp8 | 0.04054436 | ras-related GTP-binding protein C (LOC111955329), transcript variant X2, mRNA | molecular function | GTP binding | | | *Lygus hesperus* | |
| RvMc | LG3 | 4229377 | snp2 | 0.01438081 | synaptotagmin-1 (LOC111982726), transcript variant X1, mRNA | molecular function | calcium-dependent phospholipid binding | | | *Trichinella sp. T8* | |
| RvMc | LG4q.1:29 | 1710168 | snp5 | 0.003217101 | U7 small nuclear RNA (LOC111962906), ncRNA | molecular function | protein binding | | | *Homo sapiens* | |
| RvMc | LG4q.2 | 9511023 | snp7 | 0.000376754 | RAS guanyl-releasing protein 1 (LOC111963252), transcript variant X1, mRNA | molecular function | calcium ion binding | | | *Alligator mississippiensis* | |
| RvMc | LG16 | 39253610 | snp20 | 0.003394964 | tRNA methyltransferase 1 like (trmt1l), mRNA | biological process | methylation | | | *Bos indicus x Bos taurus* | |
| RvMc | LG20 | 67629341 | snp24 | 0.005706209 | protein tyrosine phosphatase, non-receptor type 13 (ptpn13), transcript variant X1, mRNA | molecular function | hydrolase activity | | | *Nothobranchius kuhntae* | |
| RvMc | LG23 | 44101810 | snp27 | 0.003521334 | sentrin-specific protease 3 (LOC111951145), transcript variant X1, mRNA | biological process | proteolysis | | | *Fukomys damarensis* | |
| RvMc | LG35 | 10370409 | snp33 | 9.50E-06 | thrombospondin type-1 domain-containing protein 7A-like (LOC111958763), mRNA | cellular component | integral component of membrane | | | *Salmo salar* | |
| RvMc | LG35 | 11632534 | snp34 | 0.01111573 | sialoadhesin-like (LOC111959068), mRNA | cellular component | membrane | | | *Alligator sinensis* | |
| RvD | LG3 | 1309919 | snp3 | 0.00016168 | plakophilin-2 (LOC111981758), mRNA | biological process | cell-cell adhesion | | | *Fukomys damarensis* | |
| RvD | LG3 | 10533240 | snp4 | 9.78E-06 | HMG box-containing protein 1-like (LOC111956082), partial mRNA | molecular function | DNA binding | | | *Scleropages formosus* | |
| RvD | LG3 | 17008289 | snp5 | 0.001299989 | collagen alpha-1(XXV) chain (LOC111951675), transcript variant X1, mRNA | cellular component | collagen trimer | | | *Amazona aestiva* | |
| RvD | LG8 | 44874795 | snp16 | 0.000103196 | piggyBac transposable element-derived protein 5 (LOC111968011), mRNA | molecular function | transposase activity | | | *Camelus dromedarius* | |
| RvD | LG18 | 19639048 | snp33 | 0.04257932 | U5 small nuclear ribonucleoprotein 40 kDa protein (LOC111977414), transcript variant X1, mRNA | biological process | cellular protein modification process | | | *Trichinella pseudospiralis* | |
| RvD | LG22 | 1712280 | snp37 | 6.14E-06 | diacylglycerol kinase delta-like (LOC111949925), mRNA | molecular function | kinase activity | | | *Hirondellea gigas* | |
| RvD | LG25 | 12700403 | snp41 | 2.17E-07 | potassium voltage-gated channel subfamily KQT member 5-like (LOC111951960), mRNA | molecular function | voltage-gated potassium channel activity | | | *Vulpes vulpes* | |
| RvD | LG26 | 36951038 | snp42 | 0.000105928 | tetraspanin-9-like (LOC111952939), mRNA | cellular component | integral component of membrane | | | *Agrilus planipennis* | |
| RvD | LG26 | 42912672 | snp43 | 2.34E-05 | no exon |  |  | | |  | |
| RvD | LG30 | 16331599 | snp46 | 0.000900496 | proteasome subunit beta type-4 (LOC111955067), mRNA | cellular component | proteasome complex | | | *Daphnia magna* | |
| RvD | LG32 | 12062945 | snp49 | 0.04257932 | MAM and LDL-receptor class A domain-containing protein 1-like (LOC111956351), mRNA | cellular component | membrane | | | *Lingula unguis* | |
| RvD | LG33 | 37189661 | snp52 | 0.000174384 | sin3 histone deacetylase corepressor complex component SDS3 (LOC111957805), transcript variant X1, mRNA | biological process | chromatin organization | | | *Pongo abelii* | |
| RvD | LG37 | 4725937 | snp57 | 0.000625001 | uncharacterized LOC111960155 (LOC111960155), mRNA | | | | | | |
| OvH | LG1 | 22712048 | snp1 | 0.01951748 | rho-related BTB domain-containing protein 3-like (LOC111962653), mRNA | Molecular function | DNA binding | | | *Clunio marinus* | |
| OvH | LG1 | 46698092 | snp2 | 0.025139 | inter-alpha-trypsin inhibitor heavy chain H3-like (LOC111968607), mRNA | biological process | hyaluronan metabolic process | | | *Scleropages formosus* | |
| OvH | LG2 | 1146747 | snp3 | 0.006525255 | dachshund homolog 1-like (LOC111973053), mRNA | biological process | microtubule-based process | | | *Aspergillus flavus* | |
| OvH | LG2 | 20546200 | snp5 | 0.004247934 | tyrosine-protein phosphatase non-receptor type 4 (LOC111976599), transcript variant X1, mRNA | Molecular function | protein tyrosine phosphatase activity | | | *Cyphomyrmex costatus* | |
| OvH | LG2 | 24564797 | snp6 | 0.001829857 | uncharacterized LOC111972302 (LOC111972302), ncRNA | | | | | | |
| OvH | LG3 | 4021307 | snp9 | 0.00767866 | nectin-1-like (LOC111980961), transcript variant X1, mRNA | cellular component | cell-cell junction | | | *Austrofundulus limnaeus* | |
| OvH | LG5 | 14562384 | snp13 | 0.02160396 | collagen alpha-2(I) chain-like (LOC111964505), mRNA | cellular component | collagen trimer | | | *Physeter macrocephalus* | |
| OvH | LG8 | 30570324 | snp18 | 0.03698202 | zinc transporter ZIP11-like (LOC111968220), mRNA | biological process | metal ion transport | | | *Tarsius syrichta* | |
| OvH | LG9 | 26888301 | snp19 | 0.000158614 | arf-GAP with SH3 domain, ANK repeat and PH domain-containing protein 2-like (LOC111968889), transcript variant X1, mRNA | Molecular function | GTPase activator activity | | | *Hirondellea gigas* | |
| OvH | LG13 | 17951333 | snp23 | 0.04335784 | neuroligin-1-like (LOC111972231), mRNA | cellular component | membrane | | | *Tropilaelaps mercedesae* | |
| OvH | LG13 | 42849796 | snp24 | 5.48E-06 | DEAH-box helicase 15 (dhx15), mRNA | Molecular function | helicase activity | | | *Cebus capucinus imitator* | |
| OvH | LG15 | 14478967 | snp26 | 0.01515163 | peptidyl-tRNA hydrolase 1 homolog (ptrh1), transcript variant X4, mRNA | Molecular function | aminoacyl-tRNA hydrolase activity | | | *Paramormyrops kingsleyae* | |
| OvH | LG17 | 36351175 | snp28 | 0.000678933 | transcription initiation factor TFIID subunit 10 (LOC111977249), mRNA | biological process | DNA-templated transcription, initiation | | | *Operophtera brumata* | |
| OvH | LG18 | 29382824 | snp29 | 0.01489883 | cilia and flagella associated protein 46 (cfap46), mRNA | biological process | axoneme assembly | | | *Chlorocebus sabaeus* | |
| OvH | LG19 | 38092254 | snp31 | 0.008020971 | ATP-binding cassette sub-family F member 2 (LOC111979175), transcript variant X2, mRNA | Molecular function | nucleotide binding | | | *Dufourea novaeangliae* | |
| OvH | LG36 | 38841524 | snp45 | 0.009419916 | NADH dehydrogenase [ubiquinone] 1 alpha subcomplex subunit 10, mitochondrial (LOC111959764), mRNA | no results | |  |  | |  |
| OvH | LG36 | 40857574 | snp46 | 0.04394857 | homeobox protein Hox-D9a-like (LOC111959924), mRNA | Molecular function | DNA binding | | | *Saccoglossus kowalevskii* | |
| DvT | LG1 | 26844658 | snp1 | 0.01951748 | receptor-type tyrosine-protein phosphatase gamma-like (LOC111963820), mRNA | molecular function | protein tyrosine phosphatase activity | | | *Ciona intestinalis* | |
| DvT | LG3 | 21737474 | snp5 | 0.006619428 | lysophospholipid acyltransferase LPCAT4 (LOC111952481), mRNA | molecular function | transferase activity, transferring acyl groups | | | *Camelus dromedarius* | |
| DvT | LG4q.1:29 | 621333 | snp7 | 0.02638717 | collagen alpha-1(XXVII) chain A-like (LOC111961212), mRNA | cellular component | collagen trimer | | | *Tropilaelaps mercedesae* | |
| DvT | LG6.1 | 10095405 | snp12 | 0.04881208 | dyslexia-associated protein KIAA0319 homolog (LOC111965041), transcript variant X1, mRNA | cellular component | membrane | | | *Tursiops truncatus* | |
| DvT | LG8 | 13864845 | snp14 | 0.002183045 | ADP/ATP translocase 2 (LOC111967456), mRNA | cellular component | membrane | | | *Labeo rohita* | |
| DvT | LG13 | 29343122 | snp20 | 0.01270873 | trafficking protein particle complex subunit 8 (LOC111971168), mRNA | cellular component | integral component of membrane | | | *Trichinella sp. T9* | |
| DvT | LG17 | 38050850 | snp28 | 0.04417134 | leucine-rich repeat-containing protein 3B-like (LOC111977062), mRNA | cellular component | integral component of membrane | | | *Salmo salar* | |
| DvT | LG20 | 4315290 | snp33 | 0.02450734 | no exon |  |  | | |  | |
| DvT | LG20 | 38658177 | snp35 | 0.01438081 | homeobox protein cut-like 1 (LOC111979971), mRNA | molecular function | DNA binding | | | *Orchesella cincta* | |
| DvT | LG23 | 44449131 | snp42 | 8.41E-06 | LHFPL tetraspan subfamily member 6 (lhfpl6), transcript variant X3, mRNA | cellular component | membrane | | | *Poecilia formosa* | |
| DvT | LG25 | 19083209 | snp45 | 0.033641 | ryanodine receptor 2-like (LOC111951725), mRNA | molecular function | calcium channel activity | | | *Salmo salar* | |
| DvT | LG26 | 20599424 | snp46 | 5.80E-06 | UDP-GlcNAc:betaGal beta-1,3-N-acetylglucosaminyltransferase 9 (LOC111952160), transcript variant X1, mRNA | molecular function | transferase activity, transferring glycosyl groups | | | *Homo sapiens* | |
| DvT | LG31 | 15348088 | snp51 | 1.22E-06 | potassium two pore domain channel subfamily K member 9 (kcnk9), mRNA | molecular function | potassium channel activity | | | *Ailuropoda melanoleuca* | |
| DvT | LG36 | 7883268 | snp55 | 1.56E-07 | ATP-dependent RNA helicase DDX18 (LOC111959622), mRNA | molecular function | hydrolase activity | | | *Acipenser ruthenus* | |
| DvT | LG36 | 33181251 | snp58 | 0.02303899 | semaphorin 5B (sema5b), mRNA | molecular function | semaphorin receptor binding | | | *Xenopus tropicalis* | |
| McvD | LG4q.1:29 | 11688130 | snp6 | 0.002693749 | WD repeat-containing protein 70-like (LOC111961442), mRNA | molecular function | threonine-type endopeptidase activity | | | *Clunio marinus* | |
| McvD | LG4q.1:29 | 12498269 | snp7 | 0.02715229 | piggyBac transposable element-derived protein 4-like (LOC111961474), mRNA | molecular function | nucleic acid binding | | | *Salmo salar* | |
| McvD | LG4q.2 | 6537561 | snp8 | 0.002151337 | chloride intracellular channel protein 4-like (LOC111963185), mRNA | biological process | chloride transport | | | *Nannochloropsis gaditana* | |
| McvD | LG6.2 | 6951300 | snp11 | 0.000370257 | cholinergic receptor nicotinic beta 3 subunit (chrnb3), mRNA | cellular component | cell junction | | | *Chlorocebus sabaeus* | |
| McvD | LG7 | 6940218 | snp12 | 4.92E-05 | helicase ARIP4 (LOC111966287), transcript variant X1, mRNA | molecular function | ATP binding | | | *Clunio marinus* | |
| McvD | LG7 | 27322574 | snp13 | 0.001463111 | prefoldin subunit 4 (LOC111966746), mRNA | biological process | protein folding | | | *Clunio marinus* | |
| McvD | LG8 | 35156854 | snp15 | 0.01354193 | regulator of G-protein signaling 9 (LOC111968233), mRNA | biological process | G protein-coupled receptor signaling pathway | | | *Charadrius vociferus* | |
| McvD | LG9 | 3762720 | snp16 | 0.03163034 | spectrin beta chain, erythrocytic (LOC111968456), transcript variant X2, mRNA | molecular function | actin binding | | | *Tinamus guttatus* | |
| McvD | LG11 | 16196149 | snp21 | 0.009419916 | potassium channel subfamily K member 15-like (LOC111970608), mRNA | molecular function | potassium channel activity | | | *Salmo salar* | |
| McvD | LG35 | 3421533 | snp43 | 0.000859749 | zinc finger protein, FOG family member 2 (zfpm2), mRNA | molecular function | RNA polymerase II transcription factor binding | | | *Pan paniscus* | |
| McvH | LG11 | 900379 | snp11 | 0.04553445 | fer-1-like protein 4 (LOC111969638), mRNA | cellular component | integral component of membrane | | | *Zootermopsis nevadensis* | |
| McvH | LG18 | 49890639 | snp18 | 0.0258357 | transmembrane protein 150A (LOC111978534), transcript variant X1, misc_RNA | cellular component | membrane | | | *Fundulus heteroclitus* | |
| McvH | LG20 | 17282628 | snp20 | 0.02450734 | sideroflexin-5 (LOC111981706), transcript variant X1, mRNA | biological process | ion transport | | | *Lygus hesperus* | |
| McvH | LG20 | 18533025 | snp21 | 0.000320021 | peripheral-type benzodiazepine receptor-associated protein 1-like (LOC111981851), mRNA | cellular component | membrane | | | *Platysternon megacephalum* | |
| McvH | LG32 | 33238578 | snp31 | 0.04881208 | kidney mitochondrial carrier protein 1 (LOC111957071), mRNA | cellular component | membrane | | | *Glycine soja* | |
| McvH | LG36 | 1352026 | snp34 | 0.01212841 | collagen alpha-1(XVIII) chain (LOC111959381), transcript variant X3, mRNA | cellular component | collagen trimer | | | *Charadrius vociferus* | |
| TvO | LG2 | 12700287 | snp6 | 0.000821596 | uncharacterized protein C7orf57 homolog (LOC111974535), transcript variant X1, mRNA | biological process | biological process | | | *Mus musculus* | |
| TvO | LG2 | 28643751 | snp8 | 0.01981823 | neurogenic differentiation factor 1 (LOC111977678), mRNA | biological process | regulation of transcription, DNA-templated | | | *Camelus dromedarius* | |
| TvO | LG3 | 159375 | snp9 | 0.000943328 | keratocan-like (LOC111955509), partial mRNA | biological process | visual perception | | | *Poecilia formosa* | |
| TvO | LG3 | 21775351 | snp12 | 0.02175842 | SPT16 homolog, facilitates chromatin remodeling subunit (supt16h), transcript variant X1, mRNA | cellular component | FACT complex | | | *Papio anubis* | |
| TvO | LG4q.1:29 | 9564341 | snp15 | 0.04444325 | retinoic acid induced 14 (rai14), transcript variant X1, mRNA | cellular component | extracellular matrix | | | *Xenopus tropicalis* | |
| TvO | LG4q.1:29 | 21717064 | snp16 | 0.004967303 | ankyrin-1 (LOC111961660), mRNA | biological process | signal transduction | | | *Camelus dromedarius* | |
| TvO | LG6.2 | 2845079 | snp19 | 0.002312779 | uncharacterized LOC111965670 (LOC111965670), transcript variant X2, ncRNA | | | | | | |
| TvO | LG6.2 | 17145963 | snp21 | 7.43E-05 | uncharacterized LOC111965565 (LOC111965565), ncRNA | | | | | | |
| TvO | LG16 | 12143852 | snp32 | 0.00056496 | uncharacterized LOC111976129 (LOC111976129), transcript variant X2, ncRNA | | | | | | |
| TvO | LG18 | 12394280 | snp35 | 0.007956303 | gap junction delta-3 protein (LOC111977403), mRNA | cellular component | plasma membrane | | | *Takifugu flavidus* | |
| TvO | LG18 | 52795929 | snp36 | 0.004360239 | bone morphogenetic protein 5 (LOC111978064), mRNA | cellular component | integral component of membrane | | | *Nothobranchius pienaari* | |
| TvO | LG20 | 41315754 | snp41 | 0.04394857 | cytospin-B-like (LOC111981317), transcript variant X3, mRNA | biological process | meiotic cell cycle | | | *Cucumis melo var. makuwa* | |
| TvO | LG22 | 9557235 | snp43 | 0.03776503 | echinoderm microtubule-associated protein-like 2 (LOC111949414), transcript variant X3, mRNA | molecular function | microtubule binding | | | *Mus musculus* | |
| TvO | LG26 | 14313045 | snp52 | 0.009217599 | basigin (LOC111952490), transcript variant X2, mRNA | cellular component | plasma membrane | | | *Camelus dromedarius* | |
| TvO | LG28 | 18150994 | snp58 | 0.0382894 | carboxyl-terminal PDZ ligand of neuronal nitric oxide synthase protein-like (LOC111954114), mRNA | cellular component | BLOC-1 complex | | | *Platysternon megacephalum* | |
| TvO | LG30 | 5859132 | snp60 | 0.02450734 | transcriptional repressor GATA binding 1 (trps1), mRNA | molecular function | metal ion binding | | | *Papio anubis* | |
| TvO | LG37 | 19080562 | snp65 | 1.70E-05 | oligosaccharyltransferase complex subunit ostc (LOC111960315), mRNA | biological process | protein glycosylation | | | *Zea mays* | |
| RvO | LG3 | 21775351 | snp5 | 0.000685037 | SPT16 homolog, facilitates chromatin remodeling subunit (supt16h), transcript variant X1, mRNA | cellular component | FACT complex | | | *Papio anubis* | |
| RvO | LG4q.1:29 | 9560336 | snp7 | 4.88E-06 | retinoic acid induced 14 (rai14), transcript variant X1, mRNA | cellular component | extracellular matrix | | | *Xenopus tropicalis* | |
| RvO | LG6.1 | 576156 | snp9 | 0.001285517 | SET domain bifurcated 1 (setdb1), mRNA | cellular component | nucleus | | | *Protopterus annectens* | |
| RvO | LG9 | 18655480 | snp11 | 0.0382894 | zinc finger protein DPF3 (LOC111968753), mRNA | molecular function | zinc ion binding | | | *Trichinella sp. T8* | |
| RvO | LG15 | 38535385 | snp14 | 0.04463678 | proline dehydrogenase 1, mitochondrial (LOC111975162), mRNA | biological process | oxidation-reduction process | | | *Dictyostelium discoideum* | |
| RvO | LG24 | 1098108 | snp26 | 0.04147309 | protein FAM19A5 (LOC111951564), transcript variant X1, mRNA | cellular component | integral component of membrane | | | *Callorhinus ursinus* | |
| RvO | LG24 | 8255688 | snp27 | 0.002151337 | vegetative cell wall protein gp1-like (LOC111951357), mRNA | molecular function | structural constituent of cuticle | | | *Diaphorina citri* | |
| RvO | LG27 | 784868 | snp28 | 0.04394857 | polyhomeotic-like protein 2 (LOC111953970), mRNA | molecular function | zinc ion binding | | | *Neomonachus schauinslandi* | |
| RvO | LG31 | 29914955 | snp31 | 0.001500516 | extensin-like (LOC111955794), mRNA | cellular component | membrane | | | *Prunus yedoensis var. nudiflora* | |
| RvO | LG36 | 2524635 | snp32 | 0.001420932 | regulator of nonsense transcripts 3A-like (LOC111959860), transcript variant X2, mRNA | biological process | nuclear-transcribed mRNA catabolic process, nonsense-mediated decay | | | *Plasmodium coatneyi* | |
| McvO | LG8 | 49368147 | snp6 | 0.006384625 | endoplasmic reticulum lectin 1 (LOC111968068), transcript variant X2, mRNA | molecular function | carbohydrate binding | | | *Lygus hesperus* | |
| McvO | LG32 | 19188679 | snp37 | 0.03698202 | uncharacterized LOC111956372 (LOC111956372), transcript variant X3, ncRNA | | | | | | |
| HvR | LG1 | 7030492 | snp1 | 0.02340302 | protein kinase C alpha type (LOC111957424), transcript variant X1, mRNA | molecular function | kinase activity | | | *Cajanus cajan* | |
| HvR | LG3 | 11599280 | snp2 | 0.003409886 | uncharacterized LOC111981471 (LOC111981471), ncRNA | | | | | | |
| HvR | LG4q.1:29 | 40302530 | snp5 | 0.001112289 | serine/threonine-protein kinase BRSK2-like (LOC111960930), mRNA | molecular function | kinase activity | | | *Tarsius syrichta* | |
| HvR | LG4q.1:29 | 44597105 | snp7 | 0.03389485 | eukaryotic initiation factor 4A-I (LOC111962020), mRNA | molecular function | nucleic acid binding | | | *Enhydra lutris kenyoni* | |
| HvR | LG13 | 43861221 | snp11 | 0.000178754 | transmembrane protein 131-like (LOC111971549), transcript variant X3, mRNA | cellular component | integral component of membrane | | | *Mesocricetus auratus* | |
| HvR | LG14 | 24062137 | snp12 | 0.000759694 | T-cell activation Rho GTPase-activating protein (LOC111973443), mRNA | molecular function | GTPase activator activity | | | *Homo sapiens* | |
| HvR | LG16 | 38346458 | snp16 | 0.04394857 | microtubule-associated serine/threonine-protein kinase 2-like (LOC111975495), mRNA | molecular function | kinase activity | | | *Mesocricetus auratus* | |
| HvR | LG19 | 12367471 | snp18 | 0.009217599 | myosin light chain 1, cardiac muscle (LOC111978968), mRNA | molecular function | calcium ion binding | | | *Corvus brachyrhynchos* | |
| HvR | LG19 | 30136314 | snp19 | 0.02188711 | choline-phosphate cytidylyltransferase A (LOC111979607), transcript variant X1, mRNA | molecular function | nucleotidyltransferase activity | | | *Camelus dromedarius* | |
| HvR | LG20 | 46736785 | snp21 | 0.01291537 | sodium/potassium-transporting ATPase subunit beta-2 (LOC111981831), transcript variant X2, mRNA | biological process | sodium ion transport | | | *Melipona quadrifasciata* | |
| HvR | LG20 | 50466081 | snp22 | 0.04394857 | transmembrane protein FAM155A-like (LOC111980313), mRNA | cellular component | integral component of membrane | | | *Lynx pardinus* | |
| HvR | LG26 | 8099691 | snp25 | 0.001184451 | ephrin-A2-like (LOC111953065), mRNA | molecular function | ephrin receptor binding | | | *Scophthalmus maximus* | |
| HvR | LG33 | 13525229 | snp30 | 0.009117259 | TBC1 domain family member 10A (LOC111957627), mRNA | molecular function | U6 snRNA binding | | | *Xenopus tropicalis* | |
| HvR | LG33 | 33323365 | snp31 | 0.01046121 | zinc finger protein 131 (LOC111957956), mRNA | cellular component | nucleoplasm | | | *Papio anubis* | |
| DvO | LG1 | 24663720 | snp2 | 0.02088757 | C->U-editing enzyme APOBEC-2 (LOC111963481), mRNA | molecular function | RNA binding | | | *Homo sapiens* | |
| DvO | LG4q.1:29 | 7162706 | snp5 | 0.02160396 | soluble lamin-associated protein of 75 kDa-like (LOC111961332), transcript variant X2, mRNA | cellular component | intermediate filament | | | *Hydra vulgaris* | |
| DvO | LG4q.2 | 21314780 | snp9 | 0.00173751 | bromodomain adjacent to zinc finger domain protein 1A (LOC111963461), transcript variant X3, mRNA | molecular function | metal ion binding | | | *Dufourea novaeangliae* | |
| DvO | LG7 | 17418987 | snp15 | 0.00373802 | DENN domain-containing protein 2C (LOC111966511), transcript variant X1, mRNA | molecular function | Rab guanyl-nucleotide exchange factor activity | | | *Homo sapiens* | |
| DvO | LG11 | 35555994 | snp23 | 0.04335784 | sodium- and chloride-dependent GABA transporter 2-like (LOC111970289), mRNA | biological process | transmembrane transport | | | *Lingula unguis* | |
| DvO | LG16 | 6530843 | snp29 | 0.00208879 | FYVE and coiled-coil domain-containing protein 1 (LOC111975752), transcript variant X2, mRNA | molecular function | metal ion binding | | | *Phytophthora nicotianae* | |
| DvO | LG16 | 15161749 | snp30 | 0.000237277 | peptidyl-prolyl cis-trans isomerase FKBP8 (LOC111975390), transcript variant X1, mRNA | molecular function | isomerase activity | | | *Macaca mulatta* | |
| DvO | LG20 | 27818780 | snp36 | 0.000387177 | kin of IRRE-like protein 3 (LOC111981964), mRNA | cellular component | integral component of membrane | | | *Melipona quadrifasciata* | |
| DvO | LG20 | 32726302 | snp37 | 1.34E-07 | opioid-binding protein/cell adhesion molecule-like (LOC111981966), transcript variant X2, mRNA | molecular function | molecular function | | | *Mus musculus* | |
| DvO | LG20 | 43606433 | snp38 | 0.033641 | protein EURL homolog (LOC111981575), transcript variant X1, mRNA | biological process | positive regulation of dendritic spine development | | | *Danio rerio* | |
| DvO | LG20 | 61024995 | snp39 | 0.001331429 | guanine nucleotide-binding protein G(q) subunit alpha (LOC111980819), mRNA | molecular function | G protein-coupled receptor binding | | | *Melipona quadrifasciata* | |
| DvO | LG24 | 9530255 | snp46 | 0.00400808 | *uncharacterized LOC111951267 (LOC111951267), ncRNA* | | | | | | |
| DvO | LG31 | 17835809 | snp53 | 0.004752522 | anillin-like (LOC111955930), transcript variant X1, mRNA | biological process | hemopoiesis | | | *Euroglyphus maynei* | |
| DvO | LG32 | 13986667 | snp54 | 0.01046388 | chromodomain helicase DNA binding protein 7 (chd7), transcript variant X1, mRNA | biological process | face development | | | *Papio anubis* | |
| DvO | LG37 | 3738783 | snp61 | 0.003123204 | transmembrane protein 256 (LOC111960416), mRNA | cellular component | integral component of membrane | | | *Strongyloides ratti* | |
| TvR | LG1 | 9981469 | snp1 | 0.04951506 | son of sevenless homolog 1 (LOC111957031), mRNA | molecular function | guanyl-nucleotide exchange factor activity | | | *Nothobranchius furzeri* | |
| TvR | LG2 | 14771845 | snp3 | 0.00573387 | serine/threonine-protein kinase tousled-like 1-B (LOC111975038), mRNA | biological process | regulation of chromatin assembly or disassembly | | | *Danio rerio* | |
| TvR | LG5 | 30381440 | snp10 | 0.000678933 | aristaless-related homeobox protein-like (LOC111964277), mRNA | molecular function | DNA binding | | | *Tropilaelaps mercedesae* | |
| TvR | LG7 | 5556872 | snp14 | 9.78E-06 | guanine nucleotide-binding protein G(s) subunit alpha (LOC111966254), transcript variant X2, mRNA | biological process | signal transduction | | | *Trachymyrmex cornetzi* | |
| TvR | LG8 | 15967402 | snp15 | 0.000915942 | no exon |  |  | | |  | |
| TvR | LG11 | 21443443 | snp20 | 0.000556339 | membrane-associated guanylate kinase, WW and PDZ domain-containing protein 3 (LOC111969958), transcript variant X3, mRNA | no results | | | |  | |
| TvR | LG15 | 46024167 | snp24 | 0.02088757 | U7 small nuclear RNA (LOC111975239), ncRNA | no results | | | |  | |
| TvR | LG17 | 25163238 | snp25 | 0.00056496 | basement membrane-specific heparan sulfate proteoglycan core protein-like (LOC111976306), mRNA | cellular component | membrane | | | *Scleropages formosus* | |
| TvR | LG18 | 18611124 | snp26 | 0.02088757 | G protein-activated inward rectifier potassium channel 1-like (LOC111977554), mRNA | molecular function | inward rectifier potassium channel activity | | | *Ictalurus punctatus* | |
| TvR | LG20 | 10018312 | snp30 | 0.01835842 | pyruvate dehydrogenase (acetyl-transferring) kinase isozyme 2, mitochondrial (LOC111981212), mRNA | biological process | carbohydrate metabolic process | | | *Rattus norvegicus* | |
| TvR | LG20 | 32008925 | snp31 | 8.41E-06 | intraflagellar transport 46 (ift46), mRNA | biological process | cilium assembly | | | *Papio anubis* | |
| TvR | LG23 | 3579517 | snp34 | 0.02231721 | glutaredoxin and cysteine rich domain containing 2 (grxcr2), mRNA | cellular component | microvillus | | | *Cebus capucinus imitator* | |
| TvR | LG25 | 1898204 | snp37 | 0.005706209 | beta-crystallin S-1-like (LOC111951602), transcript variant X2, mRNA | biological process | glycine betaine biosynthetic process from choline | | | *Burkholderia plantarii* | |
| TvR | LG25 | 11075582 | snp38 | 6.85E-06 | Golgi-specific brefeldin A-resistance guanine nucleotide exchange factor 1 (LOC111951704), mRNA | molecular function | ARF guanyl-nucleotide exchange factor activity | | | *Trichinella sp. T8* | |
| TvR | LG30 | 16691625 | snp41 | 0.002057949 | tumor necrosis factor alpha-induced protein 8-like protein 2 (LOC111955375), mRNA | biological process | regulation of apoptotic process | | | *Schistosoma haematobium* | |
| TvR | LG33 | 9156849 | snp45 | 0.02630379 | vacuolar protein sorting-associated protein 13A (LOC111957899), transcript variant X1, mRNA | molecular function | catalytic activity | | | *Talaromyces islandicus* | |
| TvR | LG33 | 32037434 | snp47 | 0.00016168 | growth arrest and DNA damage-inducible protein GADD45 gamma (LOC111957399), mRNA | biological process | regulation of cell cycle | | | *Ursus maritimus* | |
| TvR | LG36 | 21320000 | snp50 | 0.000507807 | synaptotagmin-like protein 5 (LOC111959478), transcript variant X1, mRNA | molecular function | metal ion binding | | | *Dufourea novaeangliae* | |
| MuvO | LG37 | 19080562 | snp7 | 0.02450734 | oligosaccharyltransferase complex subunit ostc (LOC111960315), mRNA | biological process | protein glycosylation | | | *Zea mays* | |
| TvMu | LG11 | 9577390 | snp2 | 0.001526367 | no exon |  |  | | |  | |
| TvMu | LG12 | 9582656 | snp3 | 0.000368374 | transmembrane protein 163 (LOC111971006), mRNA | cellular component | integral component of membrane | | | *Balearica regulorum gibbericeps* | |
| DvMu | LG33 | 10280610 | snp4 | 0.03163034 | cell surface hyaluronidase-like (LOC111958112), mRNA | biological process | viral process | | | *Duck hepatitis B virus* | |

***Suppl. Table 4.*** Additive individual lake comparisons through CMH analysis, with BLAST function, gene ontology category, based on gene ontology function.
