# Supplementary material for: Neutral and adaptive drivers of genomic change in introduced brook trout (Salvelinus fontinalis) populations revealed by pooled sequencing
Source: Ecol Evol. 2022 Feb 7;12(2):e8584. doi: 10.1002/ece3.8584 (PMC8820109; doi:10.1002/ece3.8584)
Supplement: Supplementary file 2 — Appendix S2 [file ECE3-12-e8584-s001.docx]

**Appendix B: Limnological Protocols**

Seep, inlet, and outlet data was measured by circumnavigation of each lake. A GPS point was taken at each location using a *Garmin etrex 20x*, and length measurements for each inlet/outlet/seep were taken up to 2000m from each lake (Gowan & Fausch, 1996), or until a passage barrier was reached, via GPS tracking and confirmed with Google Earth version 9.2.58.1 (Google, available online). Spawning sites were estimated by combining the number of all discernable seeps, inlets, and outlets in each lake; in rare cases where no connectivity was found, data was marked as N/A. Along each length measurement, seven equidistant transects were conducted with five flow-perpendicular sampling points, to measure: depth, substrate, and water velocity along the thalweg (Hydromatch, n.d.). Connectivity to other bodies of water, catchment area, and bathymetry was calculated using ArcGIS version 10.3.1 and obtained from Parks Canada records.

Depth profiles of temperature, dissolved oxygen, pH and conductivity were measured twice in summer in each lake at 1 m sequential depths to 0.5 m above bottom with a multiparameter YSI Professional series sonde (model 10102030; Yellow Springs Inc., Yellow Springs, Ohio, USA). HOBO MX2202 Pendant wireless temperature/light dataloggers (Onset, MA USA) were deployed at the centre of each lake, and recorded measurements every 30 minutes at 0.5 m depth from beginning of July to mid September.

Nearshore littoral macroinvertebrate communities were collected with a D-frame kick net at eight sampling stations located around the lake. Sampling was performed by the sweep method (“Kick & Sweep”) with a 500 μm “D-net” as recommended by the Ontario Benthos Biomonitoring Network (Jones et al. 2007) on a surface of approximatively 2 m^2^. Samples were concentrated with a 500-μm sieve, preserved in 95% ethanol, and they were brought back to the laboratory at the Université du Québec à Montréal (UQAM) for identification. Identification was done up to the Family level following taxonomic keys (Merrit et al. 2008; Moisan 2010). All macroinvertebrate samples were identified using SZX10 stereo microscopes (Olympus) with varying magnification (x6.3 - x10). For each sample, 100mL sub-sample was taken and counted until the 100th individual was reached. If the 100th individual was not reached within the initial 100 mL, an additional 100 mL was counted. When the 100^th^ individual was reached, the remaining part of the sub-sample was counted, and the total sub-sampled volume calculated. A ratio was then calculated between the total sub-sampled volume and the sample total volume to estimate the taxon-specific abundance of macroinvertebrates. The density of macroinvertebrates per m^2^ was averaged for the 8 samples collected per lake to obtain total macroinvertebrate density per lake.

Crustacean zooplankton were collected by whole water column vertical tows with a 35-cm diameter Wisconsin net from 0.5 m off the bottom to the surface. The zooplankton were sampled from four sampling stations along an open-water transect across each lake. The crustacean zooplankton were anaesthetized with bromoseltzer, preserved with 95% ethanol, and they were brought back to the laboratory at the Université du Québec à Montréal (UQAM) for identification. These four samples were subsequently pooled to a single sample per lake for identification and enumeration in the laboratory. The crustacean zooplankton were identified to species level with a high- resolution dissecting microscope (6.3-126x; SZ2-IL-ST; Olympus SZ) and species were enumerated for the number of individuals per L. Crustacean zooplankton were counted using a protocol that targeted mature individuals that could be identified unambiguously to species, as well as to detect rare species (Girard & Reid, 1990). Subsamples (10 mL) were taken from a standardised 50 mL sample volume, and at least 250 individuals were counted so that no more than 50 copepodids per order and no more than 30 nauplii were included in the sum to 250 individuals, even though more were counted. Taxonomic keys included Brooks (1957), Smith and Fernando (1978), De Melo and Hebert (1994), Witty (2004), Thorp and Covich (2010), Haney et al. (2013).

Lastly, a Jaccard dissimilarity index of fish species ineach lake was calculated using presence-absence data collected from the sampling period (R package Adespatial, v 0.3-8; Stéphane Dray et al., 2020).

**References**

Brooks, J. L. (1957). The systematics of North American Daphnia. *Mem Conn Acad Arts Sci. 13*:1-180.

De Melo, R. D., & Hebert, P.D. (1994). A taxonomic reevaluation of North American Bosminidae. *Canadian Journal of Zoology, 72*(10):1808-1825.

Haney, J. F., Aliberti, M. A., Allan, E., Allard, S., Bauer, D. J., Beagen, W., ... & Dufresne, J. (2013). An-Image-based Key to the Zooplankton of North America version 5.0. University of New Hampshire. *Center for Freshwater Biology* [cited 11 May 2020]. Available from http://cfb.unh.edu/cfbkey/html/

Jones, C., Somers, K., Craig, B., & Reynoldson, T. (2007). Ontario Benthos Biomonitoring Network: Protocol Manual. Dorset (ON): Ontario Ministry of Environment. *Environmental Monitoring and Reporting Branch.*

Merritt, R. W., Cummins, K. W., & Berg, M. B. (2008). *An introduction to the aquatic insects of North America. 4^th^ ed*. Dubuque (IA): Kendall Hunt publishing company.

Moisan, J. (2010). Guide d’identification des principaux macroinvertébrés benthiques d’eau douce du Québec – *Surveillance volontaire des cours d’eau peu profonds. Bibliothèque et archives nationales du Québec (CA) : Direction du suivi de l’état de l’environnement, ministère du Développement durable, de l’Environnement et des Parcs*. Smith, K. E., & Fernando, C.H. (1978). A guide to the freshwater calanoid and cyclopoid Copepoda. Crustacea of Ontario. *University of Waterloo Biology Series. 18*:1–76

Thorp, J. H., & Covich, A. P. (2010). *Ecology and Classification of North American Freshwater Invertebrates. 3^rd^ ed*. London (UK): Elsevier Academic Press.

Witty, L. M. (2004)*. Practical Guide to Identifying Freshwater Crustacean Zooplankton. 2^nd^ ed.* Sudbury (ON): Cooperative Freshwater Ecology Unit.
